# Long-chain acyl-CoA synthetases: biological functions, diseases and therapeutic targets

**DOI:** 10.1186/s43556-025-00366-4

**Published:** 2025-11-25

**Authors:** Xiaoliang Deng, Yanqun Luo, Ying Gao, Tao Wu

**Affiliations:** Institute of Interdisciplinary Integrative Medicine Research, Shanghai University of Traditional Chinese Medicine, Cailun Road, Shanghai, 201203 China

**Keywords:** Cancers, Family of ACSLs, Fatty acid metabolism, Ferroptosis, Metabolic disease, Cardiovascular and cerebrovascular diseases

## Abstract

The long-chain acyl-CoA synthetase (ACSL) family comprises key enzymes that are integral to the fatty acid metabolic pathway and play crucial roles in governing fatty acid (FA) metabolism and lipid homeostasis. These enzymes are involved in multiple pathophysiological processes, including cellular metabolism, endoplasmic reticulum stress, lipid peroxidation, and ferroptosis (iron-dependent cell death). The ACSL family consists of five isoforms—ACSL1, ACSL3, ACSL4, ACSL5, and ACSL6—each of which has been implicated in the pathogenesis, treatment, and prognosis of diverse conditions, including metabolic disorders, cancers, cardiovascular and cerebrovascular diseases, and other clinical conditions. By catalyzing the conversion of polyunsaturated fatty acids into fatty acyl-CoA, these enzymes mediate a range of biological activities, including anti-inflammatory, antioxidant, and antitumor responses, as well as the regulation of ferroptosis through lipid metabolism. Over the past five years, however, there has been a notable lack of comprehensive reviews that systematically summarize the relevance of ACSL to clinical diseases and their underlying molecular mechanisms. The present review seeks to fill this gap by summarizing recent advances in understanding the roles of the ACSL family across diverse diseases, with a focus on emerging therapeutic strategies that target these enzymes. This work provides critical insights that may inform future preclinical and clinical investigations of the ACSL family.

**Supplementary Information:**

The online version contains supplementary material available at 10.1186/s43556-025-00366-4.

## Introduction

Free fatty acids (FFAs) are categorized based on the length of their hydrocarbon chains into short-chain fatty acids (SCFAs; fewer than six carbons), medium-chain fatty acids (MCFAs; six to twelve carbons), and long-chain fatty acids (LCFAs; twelve or more carbons) [1]. Acyl-coenzyme A synthetases (ACSs) are a family of enzymes responsible for the thioesterification of fatty acids with coenzyme A, forming activated intermediates critical for lipid metabolism and the maintenance of lipid homeostasis. The product of this ACS-catalyzed reaction, acyl-coenzyme A (acyl-CoA), serves as a key substrate for complex lipid synthesis of complex lipids and for energy production via β-oxidation, protein acylation, and fatty acid-dependent transcriptional regulation [2]. The acyl-CoA synthetase long-chain(ACSL) family of enzymes plays an integral role in fatty acid (FA) metabolism [3], by facilitating FA transport and activation [4]. This activation step represents the initial and committed step in intracellular FA metabolism [5]. ACSLs primarily catalyze the conversion of FFAs to fatty acyl-CoA [6], regulating FA metabolism and maintaining lipid homeostasis [3]. In addition, they participate in various pathophysiological processes, including cellular metabolism, endoplasmic reticulum (ER) stress, ferroptosis, and tissue inflammation [6]. The ACSL family consists of five isoforms: ACSL1, ACSL3, ACSL4, ACSL5, and ACSL6 [3, 7, 8], each exhibiting distinct differ in their tissue distribution, subcellular localization, and biochemical properties [9].

In 1971, Bar-Tana et al. first isolated and purified palmitoyl-coenzyme A synthetase from rat liver microsomes, characterized its general properties and kinetic parameters, and explored its role in microsomal FA activation [10]. Subsequent studies, by the group further elucidated the enzyme's reaction mechanism and structural features [11, 12]. Hiroyuki Suzuki and colleagues were the first to clone rat ACSL cDNA, which was later identified as ACSL1, thereby marking the beginning of molecular-level research on the ACSL family [13]. In 1992, Fujino et al. identified a novel ACSL isoform in brain tissue [14]. Using bacterial functional complementation assays, Jorge M. Caviglia et al. clearly demonstrated the distinct functional roles of different ACSL isoforms using bacterial functional complementation assays. A landmark 2011 study revealed that ACSL1 specifically directs the metabolic partitioning of FA metabolism towards β-oxidation, a pivotal discovery in ACSL research [15]. In 2015, Scott J. Dixon et al. identified ACSL4 as a key mediator of ferroptosis, an iron-dependent form of cell death [16]. Hua Yuan et al. further confirmed ACSL4's central role of ACSL4 in ferroptosis, suggesting its potential as a therapeutic target for cancer (through ferroptosis induction) or neurodegenerative diseases (via ferroptosis inhibition) [17]. These findings laid the groundwork for research on ACSL and ferroptosis.

In the past three years, an increasing attention has been given to the ACSL family, particularly ACSL4 and its critical role in ferroptosis. However, a comprehensive review consolidating recent progress in ACSL research is still lacking. Current studies remain fragmented. Significant gaps in understanding persist, particularly regarding ACSL1, ACSL5, and ACSL6, whose biological functions and pathological roles remain underexplored. Moreover, the relationship between ACSL3 and ferroptosis is still poorly defined and warrants further investigation.

This review, first outlines the genetic background of the ACSL family, followed by an analysis of the protein structures and core biological functions of its members. It then summarizes the distinct roles of various ACSL isoforms in disease, incorporating evidence from both preclinical and clinical evidence. Finally, the review assesses the current state of ACSL research, identifies gaps in the literature, and provides perspectives for future research studies. By bridging gaps in previous publications, this review elucidates the mechanistic pathways linking ACSL molecules to biochemical reactions, pathophysiological processes, and disease outcomes, and synthesizes current knowledge on potential therapeutic strategies targeting the ACSL family. Ultimately, it offers a comprehensive overview of current research progress and highlights promising avenues for future exploration.

## Structure and catalytic mechanism of ACSLs

The ACSL family constitutes a crucial group of enzymes in fatty acid metabolism [3]. These broad functions are closely associated with their gene expression patterns, protein structural characteristics, subcellular localization, tissue distribution, and substrate specificity. At the genetic level, specific exonic regions of ACSL genes harbor potential binding sites that modulate downstream signaling pathway activation by regulating the binding of transcription factors or non-coding RNAs, thereby shaping cellular metabolic trajectories and stress responses. At the protein structural level, the core catalytic domains of ACSL enzymes, such as the adenosine monophosphate (AMP)-binding domain, provide the structural basis for their catalytic functions. This domain mediates interactions with the adenine and ribose moieties of ATP via conserved sequence motifs [18]. Furthermore, distinct ACSL isoforms display substantial differences in subcellular localization, tissue-specific expression, and substrate preference. These properties collectively define the functional specificity of each isoform and influence their distinct roles in metabolic disorders, cancers, and cardiovascular or cerebrovascular diseases. This section systematically elucidates the regulatory network of the ACSL family and its pathophysiological significance, tracing the progression from gene localization to protein structure and catalytic mechanisms.

### Gene related information

ACSL1 is located on chromosome 4q35.1 and consists of 31 exons [19]. ACSL3 is situated on chromosome 2q36.1 and contains 17 exons. Its promoter region contains a peroxisome proliferator-activated receptor α (PPARα) response element, which is involved in regulating the FA metabolic pathway. ACSL4 is found on chromosome Xq23 and comprises 18 exons. It shows low interspecific retention of exons, indicating functional divergence during evolution. ACSL5 is located on chromosome 10q25.2 and consists of 23 exons. Certain exons of ACSL5 include gut-specific caudal type homeobox 2 (CDX2) binding sites, which correlate with its role in intestinal FA absorption. ACSL6 is mapped to chromosome 5q31.1 and comprises 30 exons. It is evolutionarily conserved across mammals and exhibits brain-specific functional adaptations [20]. Data were obtained from the NCBI Gene database.

### Protein structure and catalytic mechanism

The genes encoding the various ACSL isoforms exhibit distinct chromosomal localization patterns, with their coding loci positioned in specific chromosomal regions, providing the genetic foundation for the functional diversity of this enzyme family. The ACSL family members share a highly conserved catalytic core structure that drives their essential enzymatic functions. However, subtle differences in the amino- and carboxy-terminal sequences, subcellular localization, and substrate-binding pockets across isoforms underlie their functional diversity. ACSL1 is a membrane-bound protein with an N-terminal transmembrane helix [9]. The primary role of the ACSL family is the conversion of LCFAs into their active form, acyl-CoAs, which are subsequently utilized in cellular lipid synthesis and β-oxidation [21]. All ACSL isozymes belong to the adenylate-forming enzyme superfamily, with their core catalytic domain being the AMP-binding domain, which mediates interaction with the adenine and ribose moieties of ATP. This structural domain mediates interactions with the adenine and ribose moieties of ATP via conserved sequence motifs (including A3, A5, A8, and A10), thereby facilitating the formation of the acyl-AMP intermediate and the efficient progression of subsequent thioester synthesis [18]. The catalytic process occurs in two steps: first, ATP reacts with the FA carboxyl group to form a fatty acyl-adenosine monophosphate (fatty acyl-AMP) intermediate; then, coenzyme A attacks this intermediate, producing acyl-CoA and releasing AMP [22, 23]. This mechanism is highly conserved across the ACSL family, ensuring consistent FA activation.

ACSL1 produces multiple transcripts across different species. ACSL3 is expressed as a single isoform. ACSL4 exists in two isoform variants, designated variant 1 and variant 2. ACSL5 includes three isoform variants: long (739 aa), short (683 aa), and exon 20-skipping (659 aa). ACSL6 has two isoform variants, V1 and V2 [24]. ACSL4 variant 1 is the predominant transcript and has been extensively characterized, while variant 2 appears to be brain-specific [25].

**Fig. 1:**
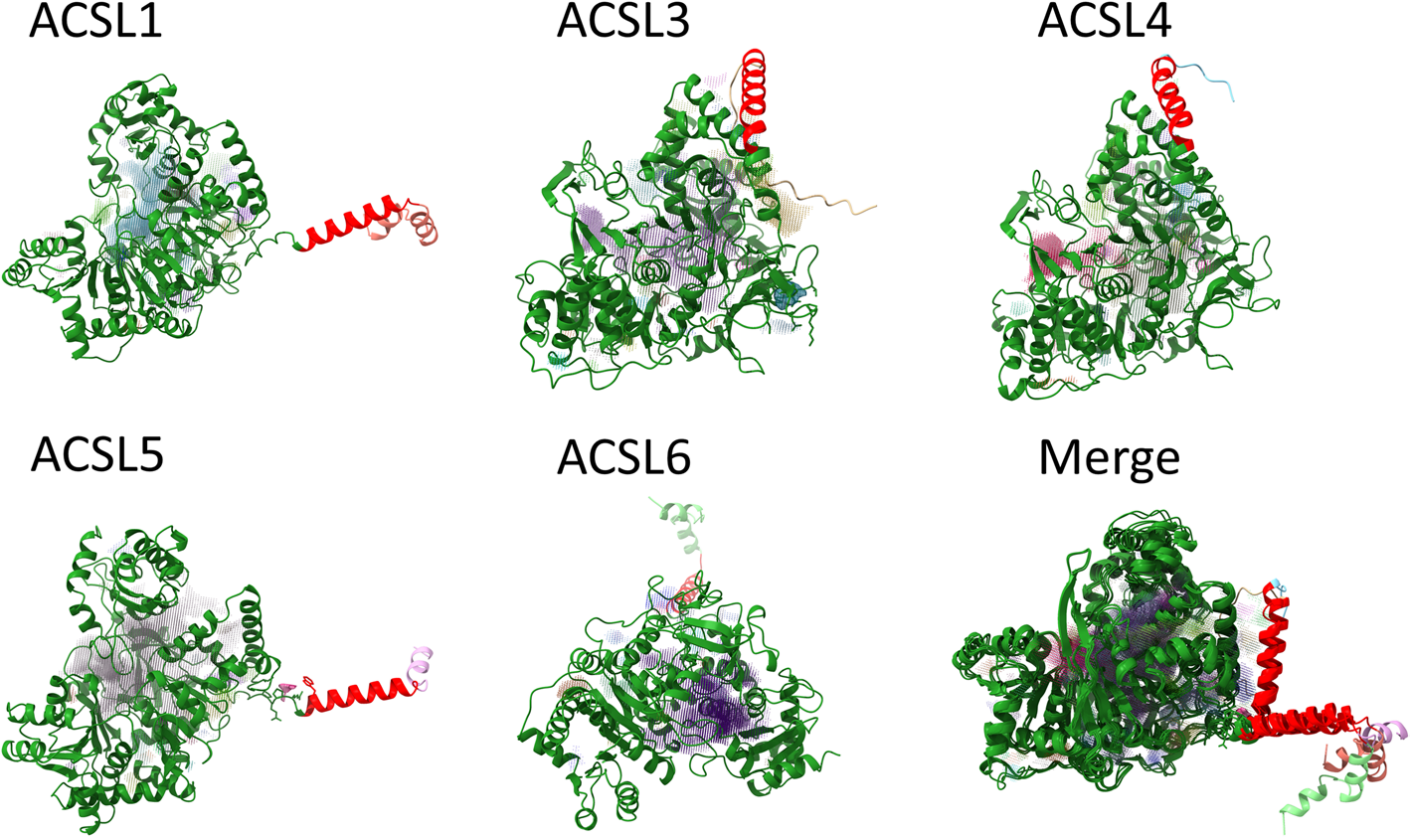
The molecular structure of ACSLs. They are virtual organism models built by computer. The detailed information about ACSLs protein sequences is in supplementary materials. ACSL: long-chain acyl-CoA synthetases

The protein sequence is provided in the Supplementary Materials, and structure of the protein is depicted in Fig. 1.

### Substrate specificity and preferences for different fatty acids

As enzymes, ACSLs exhibit distinct substrate specificities in catalyzing biochemical reactions. In addition to the common substrates, isoform-specific substrates of the ACSL family are listed in Supplementary Table 1. These data were sourced from the Rhea database (https://www.rhea-db.org/) and the UniProt database (https://www.uniprot.org/).

The ACSL family catalyzes the metabolic activation of LCFAs, with distinct substrate preferences across its isoforms. ACSL1 preferentially activates the activation of palmitoleate, oleate, and linoleate. ACSL3 primarily activates myristate, laurate, arachidonate, and eicosapentaenoate. ACSL4 is preferentially involved in the activation of highly unsaturated fatty acids (HUFAs), including docosahexaenoic acid (DHA), adrenic acid, eicosapentaenoic acid (EPA), and arachidonic acid (AA) [25, 26]. ACSL5 activates the activation of a broad spectrum of saturated FAs, with a particular preference for C16–C18 unsaturated FAs. The preferred substrates of ACSL6 is DHA. It plays a pivotal role in the nervous system.

### Tissue-specific and subcellular localization

**Fig. 2:**
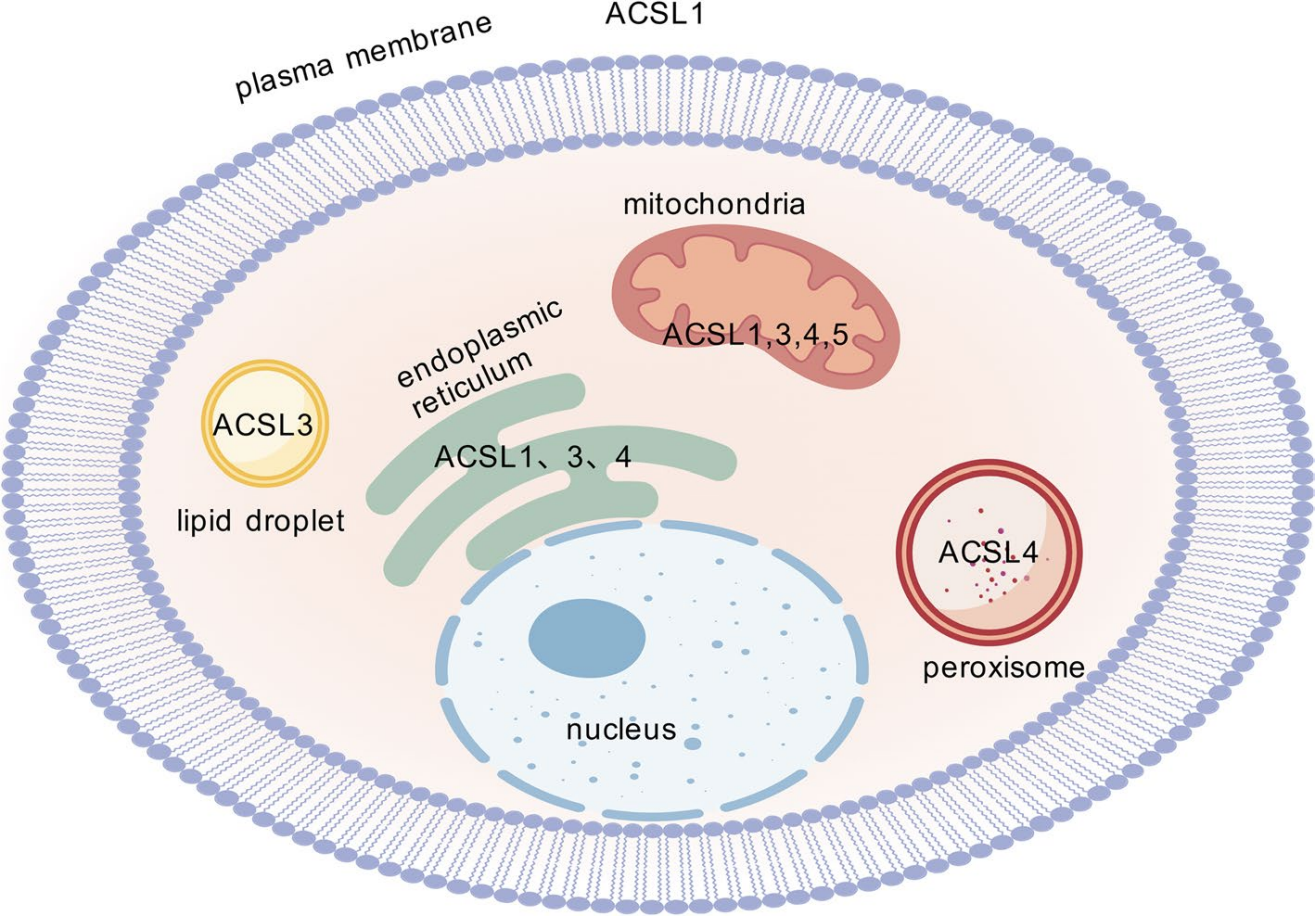
The Subcellular localization of ACSLs. Members of the ACSL family exhibit distinct subcellular localizations, which influence their specific functions in fatty acid metabolism and other cellular processes. ACSL: long-chain acyl-CoA synthetases

Subcellular localization and tissue-specific expression are critical determinants of the functional diversity among the subtypes of this protein family. This section systematically summarizes the distribution patterns of their different subtypes. Each ACSL isoform displays unique tissue distribution and subcellular localization. ACSL1 is predominantly overexpressed in hepatic and adipose tissues. It is localized to the outer mitochondrial membrane [27], the plasma membrane, and the ER [9, 28]. ACSL3 is highly expressed in the brain and prostate gland [7, 29], and it localizes to the mitochondria, ER, and lipid droplets (LDs) within the cell [5, 12]. ACSL4 is expressed in the adrenal gland, ovaries, testes, and brain, with localization to the ER, peroxisomes, and mitochondria-associated membranes [13]. ACSL5 is primarily localized to the mitochondria [30] and is widely expressed in the small intestine, liver, spleen, uterus, skeletal muscle, and adipose tissue [31, 32]. ACSL6 is highly abundant in the cerebrum and testes [33], although its subcellular localization remains unidentified [30]. The subcellular localization of the ACSL isoforms is illustrated in Fig. 2.

Basic information regarding the chromosomal localization, substrate preference, subcellular localization, and other characteristics of the ACSL family of genes and proteins is summarized in Table 1.

**Table 1:** The chromosomal localization, substrate preference, subcellular localization of ACSLs

Isoenzyme	chromosomes	exons	Preferred fatty acids	Subcellular localization	Organizational positioning	Catalytic reaction
ACSL1	4q35.1	31	palmitoleate, oleate and linoleate	outer mitochondrial membranes, plasma membrane, ER	hepatic and fat tissues	long-chain fatty acid + ATP + CoA = long-chain fatty acyl-CoA + AMP + diphosphate
ACSL3	2q36.1	17	Preferentially uses myristate, laurate, arachidonate and eicosapentaenoate as substrates	mitochondria, ER, and lipid droplets	cerebrum and prostate	
ACSL4	Xq23	18	Preferentially activates arachidonate and eicosapentaenoate as substrates	ER, peroxisomes, mitochondria-associated membranes	adrenal gland, ovaries, testes, and brain	
ACSL5	10q25.2	23	Utilizes a wide range of saturated fatty acids with a preference for C16-C18 unsaturated fatty acids	mitochondria	small intestine, liver, spleen, uterus, skeletal muscle, adipose tissue	
ACSL6	5q31.1	30	docosahexaenoic acid	unknown	cerebrum, testicular	

*ACSL* long-chain acyl-CoA synthetase, *ER* endoplasmic reticulum

## Biological functions of ACSLs

Defining the functional characteristics of proteins provides a critical foundation for related research, as it not only elucidates the specific mechanisms by which proteins contribute to disease onset and progression but also establishes the theoretical basis for subsequent target identification and preclinical investigation. In the case of the ACSL family, analyzing gene exon features, structure-function domains, substrate preferences, subcellular localization, and tissue-specific expression enables a systematic interpretation of both the shared and unique functional characteristics among its members. As an essential class of lipid-metabolizing enzymes, the ACSL family shares a central role in lipid metabolism. Moreover, differences in substrate specificity and subcellular localization among family members confer distinct biological functions on each enzyme.

### Canonical function: channeling fatty acids into metabolic pathways

Members of the ACSL family share a conserved set of fundamental biological functions. However, each isoform exhibits distinct functional characteristics based on its structural and biochemical properties. The ACSL family catalyzes the conversion of FFAs into fatty acyl-CoA, as outlined previously. Once activated by ACSL to form fatty acyl-CoA, FAs become central metabolic intermediates, which can be directed into various pathways based on the cellular energy status and metabolic needs. The enzymatic activity and subcellular localization of ACSL enzymes modulate the flux of fatty acyl-CoA, influencing metabolic outcomes. ACSL enzymes are involved in several key metabolic pathways, including β-oxidation [21], triglyceride (TG) synthesis, phospholipid (PL) synthesis [3, 9] and signal transduction. Different ACSL isoforms exhibit unique subcellular localizations and tissue distributions, reflecting their specialized roles. In mammals, multiple ACSL isoforms are expressed. ACSL1 is primarily localized to the mitochondria-associated ER membrane, where it plays a key role in β-oxidation [15, 21]. It is anchored to the mitochondrial outer membrane via an N-terminal region that interacts with carnitine palmitoyltransferase 1b (CPT1b) in C2C12 myotubes [27]. ACSL1 promotes FA β-oxidation, generating acyl-CoA and ATP to support cellular survival and proliferation [34]. It also mediates lipotoxicity induced by the saturated FA palmitate [35] and is essential for uterine remodeling, protecting stromal cells from palmitate-induced stress [36]. ACSL3 and ACSL4 are localized to the ER and peroxisomes, organelles primarily involved in lipid synthesis. ACSL4 is critical for normal steroid hormone biosynthesis [37] and contributes to maintaining membrane fluidity of cellular membranes [38]. Additionally, ACSL4 promotes endometrial placentalization by activating the β-oxidation pathway [39]. ACSL5 catalyzes the formation of acyl-CoA in the cytoplasm using LCFAs (C16-C20) as substrates. These acyl-CoAs are subsequently utilized for the complex lipid synthesis or transported into mitochondria for β-oxidation [1]. ACSL6 preferentially directs FAs toward the TG and PL synthesis, playing a vital role in both FA metabolism synthesis and storage [40].

The lipid metabolic pathways involving ACSLs also feed into critical downstream processes. For instance, ferroptosis, an iron-dependent form of cell death, is closely linked to lipid peroxidation, which is initiated by the metabolism of PUFAs. The fundamental functions of ACSL isoforms are intrinsically linked to their expression patterns and subcellular localization. The metabolic intermediates produced by ACSL enzymes serve as key entry points into diverse functional pathways. The following section will provide an in-depth discussion of these pathways and the isoform-specific functional properties of ACSLs.

### Non-canonical isoform-specific functions

Beyond their established roles in metabolism, ACSL isoforms are also involved in atypical functions, including modulation of inflammatory responses, lipid peroxidation, protein palmitoylation, cell signaling, and organelle dynamics. Each ACSL isoform selectively influences distinct pathophysiological processes.

ACSL1 is primarily regulates cell signaling and mediates inflammatory responses [41], with a noted involvement in ferroptosis. Its role in the inflammatory response may be mediated through PPARα and PPARγ signaling pathways [42]. Specific knockdown of ACSL1 in macrophages reduces lysosomal damage and inflammasome activation in response to lipid overload [43]. In addition, inhibition of ACSL1 markedly suppresses lipopolysaccharide (LPS)-induced granulocyte–macrophage colony-stimulating factor (GM-CSF) production, and through its interaction with the mitogen-activated protein kinase (MAPK)/nuclear factor kappa-light-chain-enhancer of activated B cells (NF-κB) signaling pathway, ACSL1 contributes to the development of chronic low-grade inflammation [44]. Regarding ferroptosis, ACSL1 mediates α-eleostearic acid (αESA)-induced cell death by facilitating the incorporation of αESA into neutral lipids, including triacylglycerols. Inhibition of triacylglycerol synthesis suppresses αESA-induced ferroptosis [45]. Additionally, ACSL1 has been reported to exert anti-ferroptotic effects primarily through the ferroptosis suppressor protein 1/coenzyme Q10 (FSP1/CoQ10) pathway [46].

ACSL3, which is predominantly involved in ferroptosis regulation, preferentially activates monounsaturated fatty acids (MUFAs) and inhibits ferroptosis [47]. Its expression is positively correlated with LD accumulation; downregulation of ACSL3 reduces LD formation, while its overexpression promotes LD accumulation. ACSL3 also enhances FA activation. In breast cancer (BC) cells overexpression of ACSL3 reduces FFA levels, whereas its downregulation increases FFA levels [48]. LCFA metabolism tightly regulates ferroptosis. In this process, ACSL4 acts as a potent promoter, whereas ACSL3 is primarily associated with ferroptosis resistance [49].

ACSL4 plays a critical role in ferroptosis [50] and in regulating inflammatory responses. It serves as a context-dependent regulator of ferroptosis [51]. As key enzyme in ferroptosis-related lipid metabolism, ACSL4 activates PUFAs to form PUFA-CoA, which is subsequently esterified into PUFA-containing PLs (PUFA-PLs) [52]. By enriching cellular membranes with PLs prone to peroxidation, ACSL4 promotes ferroptosis [53, 54]. ACSL4 knockout (ACSL4KO) cells exhibit significantly increased resistance to ferroptosis [55], and the enzyme is considered a potential therapeutic target in cancer [56]. ACSL4 facilitates the production of lipid-derived reactive oxygen species (ROS) during ferroptosis by regulating FA metabolism [57]. ACSL4 deficiency inhibits the incorporation of PUFAs into membrane PLs, effectively preventing ferroptosis [58, 59]. Conversely, ACSL4 overexpression increases cellular susceptibility to ferroptosis [57]. Clinical studies have investigated it as a potential marker of ferroptosis (ClinicalTrials.gov, Identifier: NCT06890741, https://trialsearch.who.int/, Identifier: CTRI/2024/12/077831, ITMCTR2024000136, https://www.chictr.org.cn/, Identifier: ChiCTR2400086119). Regarding its role in inflammation, ACSL4 dysfunction has been linked to enhanced inflammatory responses through the exacerbation of the eicosanoid storm [60]. Chronic ACSL4 deficiency in resident peritoneal macrophages (rpMACs) reduces AA incorporation into PLs, thus decreasing lipid mediator synthesis and attenuating inflammation [61]. ACSL4 also plays a critical role in regulating mitochondrial function and FA metabolism in dendritic cells (DCs). ACSL4 inhibition reduces FA oxidation (FAO), increases AA levels, and decreases acyl-CoA synthesis in DCs, while increasing mitochondrial superoxide production (MitoSOX) in DCs, leading to mitochondrial rupture, vacuolization, and cristae thinning [62]. ACSL4 has been implicated in neuroinflammation and systemic inflammatory responses [63], and its expression is associated with cancer prognosis through modulation of immune pathways [64].

In addition to its role in lipid metabolism, ACSL5 is primarily involved in inflammatory responses and cellular energy metabolism, in addition to its role in lipid metabolism. Study has confirmed that ACSL5 influences tumor progression via immune pathways [65]. ACSL5 functions as an immune-dependent tumor suppressor, enhancing tumor sensitivity to programmed cell death protein 1 (PD-1) blockade therapy and promoting CD8^+^ T cell-mediated cytotoxicity in vivo and in vitro through regulation of major histocompatibility complex class I (MHC-I)-mediated antigen presentation [65]. One study also reported that ACSL5 acts as a downstream effector of interferon in regulating cellular energy metabolism [66]. Despite these insights, significant gaps remain in understanding ACSL5's involvement in atypical pathways beyond lipid metabolism, representing a promising area for future research.

Due to its specificity for DHA, ACSL6 plays critical physiological roles in visual function and in the nervous and reproductive systems [33]. ACSL6 is the primary enzyme driving DHA enrichment in spermatogenic cells and is essential for normal spermatogenesis and male fertility [67]. Knockdown of ACSL6 impairs normal spermatogenesis [68]. In the retina, ACSL6 facilitates the local enrichment of di-DHA and ultra-long-chain PUFA (ULC-PUFA)-containing PLs, supporting normal visual function and retinal homeostasis [69]. Although ACSL6 has also been suggested to play a role in cancer; research on this topic remains limited. Investigating the link between DHA specificity and cancer development presents an important avenue for future study.

## Pathophysiological roles of ACSLs in diseases

The pivotal role of the ACSL family in lipid metabolism [3, 9] and inflammatory responses [41] underlies its influence on the development of metabolic disorders and cardiovascular or cerebrovascular diseases. Recent studies have identified crucial roles of ACSL1, ACSL3, and ACSL4 in ferroptosis [45, 49, 58, 59], thereby opening new avenues for exploring their involvement in tumorigenesis and cancer therapy. Concurrently, the unique binding affinity of ACSL6 for DHA confers distinctive importance in elucidating the pathological mechanisms of neurological disorders and developing potential therapeutic interventions [33].

Notably, different ACSL isoforms further enhance their therapeutic potential through participation in the regulation of multiple signaling pathways. These pathways include, but are not limited to, those involved in lipid metabolism, inflammatory signaling, and cell death, providing novel insights for the development of subtype-specific targeted therapies.

This section systematically summarizes recent research advances on the ACSL family across diverse disease contexts, with a focus on elucidating the molecular mechanisms and functional characteristics of individual isoforms in specific pathological conditions. This approach provides a comprehensive perspective that deepens our understanding of the pathophysiological functions of the ACSL family. By integrating existing research findings, this work aims to establish new theoretical foundations for future diagnostic and therapeutic strategies targeting ACSL-related diseases.

### Metabolism-related diseases

#### Metabolic dysfunction-associated steatotic liver disease

The nomenclature of fatty liver disease has undergone a significant paradigm shift. The previous terms, nonalcoholic fatty liver disease (NAFLD) and nonalcoholic steatohepatitis (NASH), which were based on exclusion criteria, have been replaced by metabolic dysfunction-associated fatty liver disease (MAFLD) and metabolic dysfunction-associated steatohepatitis (MASH). ACSL1 plays a critical role in MAFLD. Research indicates that mitochondrial FAT/CD36 functions acts as a molecular bridge between LCFAs and ACSL1, enhancing long-chain acyl-CoA production, which promotes FAO and prevents lipid accumulation and ROS overproduction in hepatocytes [70]. ACSL1 deletion suppresses the expression of several key enzymes involved in bile acid biosynthesis, leading to reduced hepatic bile acid levels and altered bile acid composition [71], providing a foundation for further exploration. ACSL4 expression is elevated in patients with MASLD [72], where it plays a pivotal role in hepatic lipid metabolism and is dysregulated in this condition [73]. Mechanistically, ACSL4 inhibition has been shown to reduce lipid accumulation by enhancing mitochondrial respiration, promoting hepatocyte FA β-oxidation, and upregulating PPARs [74]. Knockdown of ACSL4 attenuates high-fat diet (HFD)-induced hepatic steatosis and fibrosis [73]. For instance, inhibition of ACSL4 using rosiglitazone or ACSL4 siRNA significantly mitigated arsenic-induced MASH and ferroptosis by reducing 5-hydroxyeicosatetraenoic acid (5-HETE) levels [75]. Moreover, downregulation of ACSL4 has been shown to alleviate MAFLD by inhibiting ferroptosis [76, 77]. At the molecular level, hypomethylation of specific CpG sites in the ACSL4 promoter has been associated with an increased risk of MASH [78], offering valuable insights into the potential for targeting ACSL4 in MASLD and MASH treatment. ACSL1 has also been implicated in fatty liver development by promoting FA uptake [79]. In addition to this, ACSL1 associates with mitochondria via TANK (TRAF family member-associated NF-κB activator)-binding kinase 1 (TBK1) and participates in FAO. Inhibition of TBK1, which impairs ACSL1 activity, has been shown to alleviate fatty liver [80]. In addition to ACSL1 and ACSL4, ACSL5 has been identified as a target of SIRT6, which alleviates MAFLD by promoting FAO [81].However, research on the role of ACSL5 in MAFLD is still limited. Studies investigating ACSL3 and ACSL6 in MAFLD are virtually nonexistent, representing a promising avenue for future research, especially considering ACSL3's role of ACSL3 in ferroptosis in contrast to ACSL4.

#### Diabetes

The ACSL family plays a pivotal role in the pathogenesis of diabetes and its associated complications. Elevated ACSL1 expression in macrophages has been shown to exacerbate inflammatory responses and disease severity in diabetic patients. Knockdown of ACSL1 in macrophages reduces lipid accumulation under diabetic conditions [82]. Yang et al. demonstrated that depletion of Sortilin increases ACSL1 levels, alleviating obesity by inhibiting the degradation of ACSL1 from mitochondria to lysosomes [83]. Currentt evidence, suggests that targeting ACSL1 could be a potential therapeutic strategy for atherosclerosis in diabetic patients. In addition to ACSL1, ACSL4 has been implicated in diabetes and complications. One study revealed that ACSL4 modulates glucose-induced insulin secretion in pancreatic islet cells [73]. Furthermore, ferroptosis, partially in part by ACSL4, may serve as a potential therapeutic target for type 2 diabetes mellitus (T2DM). Ferroptosis is linked to impaired insulin secretion in pancreatic β-cells, which are particularly vulnerable to this form of cell death. Monitoring ferroptosis-related factors may aid in managing T2DM, where ACSL4 plays a significant role [84]. ACSL4-driven ferroptosis, is primarily driven by lipid remodeling. Jiang et al. suggested that m5C modification of ACSL6 might contribute to metabolic alterations in diabetes [85]. These findings highlight ACSL1 and ACSL4 as promising therapeutic targets for diabetes, with their mechanisms closely related to lipid metabolism, lipid peroxidation, inflammation, and ferroptosis.

The ACSL family is also involved in various diabetic complications. Diabetic nephropathy, a major complication of diabetes has been linked to ACSL1. Wang et al. demonstrated that ACSL1 contributes to the development of diabetic nephropathy by modulating lipid metabolism and inflammatory responses through PPARα and PPARγ signaling, ACSL1 knockdown was shown to ameliorate disease progression [42]. Conversely, reduced expression of protein arginine methyltransferase 6 (PRMT6) expression promotes lipid peroxidation and PL-PUFA synthesis by upregulating ACSL1, thereby exacerbating ferroptosis and accelerating diabetic nephropathy progression [86]. A cross-sectional study reported ACSL4 expression and the progression of diabetic nephropathy [87], with the effect potentially mediated by ferroptosis [88]. ACSL5, another ACSL family isoform, exacerbates diabetic nephropathy by upregulating FA transport protein 2 (FATP2), which promotes apoptosis in lipid-laden adipocytes [89]. Beyond diabetic nephropathy, the ACSL family is implicated in other diabetic complications. Elevated glucose levels may promote diabetic retinopathy through ACSL4 [90]. AlkB homolog 5 (ALKBH5) has been shown to alleviate diabetic retinopathy by decreasing m6A modification of ACSL4 mRNA, thus attenuating ferroptosis [91]. Endothelial dysfunction a common complication in diabetes, is influenced by ACSL4. NKAα1 promotes the autophagic degradation of ACSL4 via the lysosomal pathway, inhibiting ferroptosis and oxidative stress in endothelial cells, ultimately preventing diabetic endothelial dysfunction [92].

#### Obesity

An HFD induces upregulation of ACSL1. Downregulation of ACSL1 enhances insulin sensitivity and glucose uptake by reducing long-chain acyl-CoA (LCA-CoA), ceramide (Cer), and diacylglycerol (DAG) levels, thereby alleviating obesity [93]. In contrast, ACSL4 plays a paradoxical role in obesity. It regulates plasma TG, glucose metabolism, and hepatic PL synthesis. ACSL4KO induces an insulin-resistant phenotype, while partial downregulation of ACSL4 protects against HFD-induced p53 activation and its downstream pro-inflammatory signaling [94]. ACSL4 overexpression has also been shown to reverse the effects of traumatic acid (TA) [95]. Further research is needed to fully understand the effects of ACSL4 upregulation and downregulation and to clarify the mechanisms that mediate its beneficial or detrimental effects following knockdown. ACSL5 is predominantly expressed in small intestinal epithelial cells [96]. In ACSL5 knockout mice, fasting blood glucose levels decrease, insulin sensitivity improves, and energy expenditure increases [97]. These metabolic effects are associated with genetic polymorphisms in ACSL5 [98]. Mechanistically, ACSL5 overexpression modulates systemic metabolism by influencing FA β-oxidation, altering mitochondrial respiratory capacity, impairing insulin signaling, and increasing mitochondrial oxidative stress [99]. Conversely, ACSL5 knockdown reduces food intake by increasing glucagon-like peptide (GLP) and peptide YY (PYY) secretion and decreasing gastric emptying rate, mitigating HFD-induced obesity and insulin resistance [96]. Collectively, these findings suggest that ACSL5 may serve as a potential therapeutic target for obesity and hyperlipidemia. However, unresolved questions remain, including whether targeting intestinal ACSL5 and modulating endogenous enteroendocrine hormones elicit sex-specific effects [96]. Jung et al. demonstrated that ACSL6 contributes to glucose and FA cycling by activating FA anabolic pathways that regulate FA re-esterification in skeletal muscle, and that ACSL6 knockdown improves metabolic outcomes [100]. Significant gaps remain in the investigation of ACSL6 in obesity, representing a promising area for further research.

**Fig. 3:**
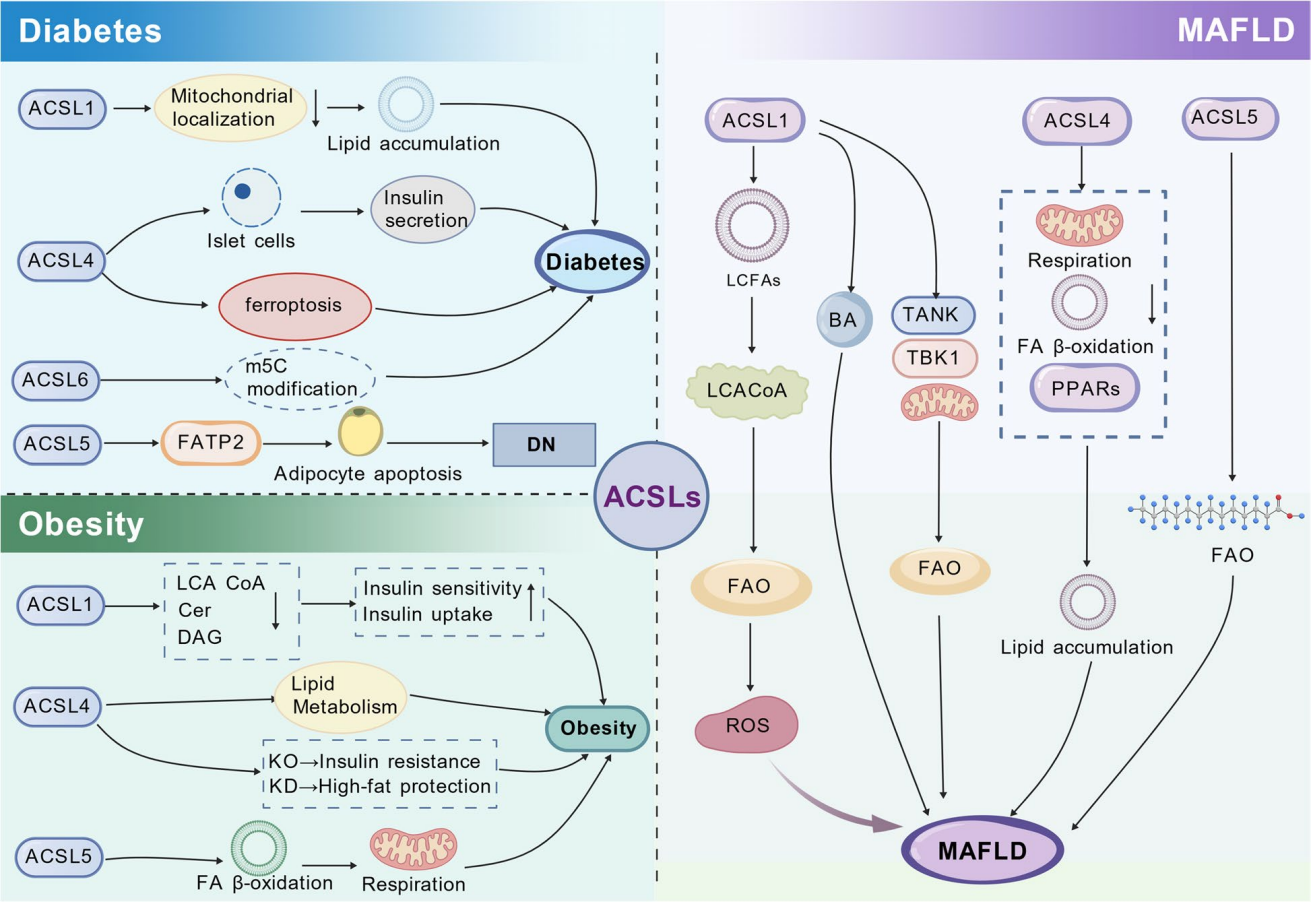
The mechanisms of the ACSL family in MAFLD, obesity, and diabetes. This figure depicts the mechanistic pathways through which ACSLs contribute to the development and progression of metabolic diseases. ACSL (long-chain acyl-CoA synthetases), FATP2 (fatty acid transport protein 2), DN (diabetic nephropathy), LCA-CoA (long-chain acyl-coenzyme A), Cer (ceramide), DAG (diacylglycerol), KO (knockout), KD (knockdown), FAO (fatty acid oxidation), ROS (reactive oxygen species), BA (bile acid), TANK (TRAF family member-associated NF-κB activator), TBK1 (TANK-binding kinase 1), FA (fatty acid)

Based on the findings summarized in this section, the role of the ACSL family in metabolic diseases is illustrated in Fig. 3. ACSLs primarily exert their effects in metabolic diseases by regulating lipid metabolism and ferroptosis.

### Cancer

The ACSL family represents a potential target for cancer treatment. Recent studies have highlighted the role of ACSL4 in ferroptosis within cancer cells, suggesting that ACSL4 may promote cell proliferation and invasion, while also sensitizing cancer cells to ferroptosis [30]. Many cancer cells’ sensitivity to ferroptosis is limited by the absence of ACSL expression [101], positioning it as a potential target for tumor therapy and a biomarker for cancers [56]. ACSL4 has been shown to promote tumor cell metastatic extravasation and colonization by enhancing plasma membrane fluidity [102]. Yu, Sun et al. found that ACSL4 may influence cancer prognosis by interacting with aggressive immune cells [64]. Furthermore, enhancing tumor apoptosis through ACSL4 inhibition and targeted acetylation of signal transducer and activator of transcription 3 (STAT3) was associated with a reduction in mitochondrial membrane PLs, providing a critical strategy to overcome cancer cell chemoresistance [103]. ACSL4-mediated ferroptosis is influenced by the immune and metabolic environment, making it a potential target for anti-tumor immunotherapy [104]. ACSL1 has also been identified as a potential target in cancer treatment, particularly due to its unique role of ACSL1 in immune-related factors, suggesting its value as a therapeutic target [105]. Additionally, ACSL1 may serve as a potential target for cachexia therapy [106]. Along with ACSL4 and ACSL1, ACSL5 has also been considered an immune-dependent cancer suppressor gene. It exerts this function by inhibiting NOD-like receptor family CARD domain-containing 5 (NLRC5), which impairs MHC-I presentation [65]. In addition to ACSL1, ACSL4, and ACSL5, ACSL3 and ACSL6 also contribute to cancer progression, with their roles to be further elaborated in the context of specific cancers.

#### Lung cancer

The ACSL family has been implicated in lung cancer, with elevated expression of ACSL3 and ACSL4 observed in this disease [107]. ACSL1 has been identified as a potential biomarker for NSCLC, acting as a protective factor [24]. In lung adenocarcinoma, ACSL3 is upregulated in tumor tissues, and its silencing induces lipid peroxidation and ferroptosis in lung cancer cells, suggesting potential therapeutic benefits [108]. A recent study demonstrated that ACSL3 knockdown enhances the efficacy of immunotherapy in vivo and promotes the development of an anti-tumor immune microenvironment. These findings provide valuable insights for clinical translation, with the combination of ACSL3 knockdown and anti-PD-1 therapy potentially preventing lung adenocarcinoma progression by increasing interferon-α (IFN-α) secretion, enhancing CD8^+^ T-cell infiltration and reducing immunosuppressive M2-like macrophages [109]. In addition to ACSL3, ACSL4 has been shown to co-regulate ferroptosis in lung adenocarcinoma in conjunction with lysophosphatidylcholine acyltransferase 3 (LPCAT3) and yes-associated protein (YAP) [110, 111]. The sensitivity of many tumor cells to ferroptosis is limited by low or absent ACSL4 expression [101]. In NSCLC, ACSL4 expression is markedly reduced in cell lines, with clinical studies indicating that higher ACSL4 expression correlates with improved prognosis in patients with NSCLC. Mechanistically, ACSL4’s effects are closely associated with ferroptosis, as outlined previously. Here, ACSL4 promotes ferroptosis by increasing lipid peroxidation, thereby enhancing anti-tumor efficacy in NSCLC [112]. Radiation therapy-induced upregulation of ACSL4 has been shown to enhance lipid synthesis and oxidative stress, to promote ferroptosis and modulating ROS levels in tumor cells following irradiation [113]. These findings suggest that ACSL4 agonists may serve as potential adjuvants in radiation therapy. Beyond ACSL3 and ACSL4, ACSL5 has been reported to influence lung cancer cell behavior by modulating the effects of lysophosphatidylcholine through the remodeling of transcriptional regulators [114]. ACSL5 also plays a critical role in inhibiting cell proliferation by regulating the metabolism of palmitic and stearic acids [115]. Although ACSL1 is expressed in lung tissue, significant gaps remain in understanding its role in lung cancer development, making it a promising target for future investigation.

#### Liver cancer

In hepatocellular carcinoma (HCC), ACSL3 and ACSL4 have been extensively studied, with several studies indicating differential expression of these enzymes in normal liver tissue, metastatic liver lesions, and HCC tissues. These enzymes hold potential as adjunctive diagnostic markers and therapeutic targets [116]. Mechanistically, ACSL4 has been shown to activate de novo lipogenesis by upregulating adipogenic enzymes. ACSL4 enhances lipogenic enzyme expression through the cellular myelocytomatosissterol regulatory element-binding protein 1/(c-Myc/SREBP1) signaling pathway. SREBP1-mediated adipogenesis being crucial for ACSL4-driven HCC cell growth and metastasis in vivo [117]. ACSL4-mediated ferroptosis has emerged as a key target for both the progression and treatment of HCC [118]. Furthermore, ACSL4 modulation via deubiquitination has been demonstrated to promote HCC by influencing FA biosynthesis, lipid peroxidation, and the tumor immune microenvironment [119]. ACSL4 also remodels the lipid profile in hepatocytes adjacent to tumors by increasing PUFA-containing lipids, which induces hepatocyte senescence and subsequently promotes tumor progression [120]. The expression of ACSL4 is associated with a poorer prognosis in HCC. One study showed that silencing ACSL4 improved HBV-related HCC by upregulating farnesoid X receptor (FXR) levels, which reduced bile acid levels and inhibited M2 macrophage polarization [121]. Beyond HCC, ACSL4 is highly expressed in hepatoblastoma and is considered a key gene in the proliferative subtype of this cancer [122].

ACSL3 expression has also been linked to poor survival outcomes [123]. ACSL3 promotes the synthesis of 1-palmitoyl-2-oleoyl-sn-glycero-3-phosphocholine (POPC), which activates the PPARα pathway and upregulates downstream lipid metabolism genes. This process facilitates HCC growth and metastasis through enhanced lipid catabolism and anabolism [124]. Elevated ACSL3 expression inhibits ferroptosis in HCC cells and contributes to sorafenib resistance in patients [125, 126].

In addition to ACSL3 and ACSL4. ACSL1 and ACSL6 have also been studied in HCC. ACSL6 is phosphorylated at Ser674 by extracellular signal-regulated kinase 2 (ERK2), and this phosphorylation subsequently interacts with IL18R to modulate NF-κB gene expression, thereby promoting tumor progression [127]. ACSL1 may serve as a potential therapeutic target in HCC by modulating neo-lipid synthesis and FA β-oxidation, but the precise mechanisms remain to be fully elucidated.

#### Colorectal cancer

ACSL3 is upregulated in colorectal cancer (CRC) tissues, where it activates FAO pathways both in vivo* and vitro*, and plays a critical role in epithelial-mesenchymal transition (EMT), invasion, and metastasis of CRC cells [128]. Stabilization of ACSL3 mRNA promotes ferroptosis in CRC cells, acting as a tumor-suppressive mechanism [129]. Elevated methylation of ACSL4 promotes ferroptosis in CRC cells [130]. In addition to regulating ferroptosis, ACSL4 modulates immune mechanisms. Upregulation of ACSL4 inhibits the retinoic acid-inducible gene I-mitochondrial antiviral-signaling protein (RIG-I-MAVS) pathway, leading to decreased interferon-beta (IFN-β) production, impaired T-cell chemotaxis, and ultimately promoting tumor immune escape and progression [131]. Ubiquitin-mediated degradation of ACSL4 can make tumors resistant to ferroptosis, correlating with poor prognosis. Conversely, ACSL4 downregulation inhibits lipid peroxidation and ferroptosis in CRC cells by suppressing PUFA biosynthesis, thus increasing resistance to PD-1 therapy [132]. ACSL1 has also been suggested to contribute to CRC progression via the ferroptosis pathway [133], though the precise mechanism remains unclear.

#### Breast cancer

BC has been associated with ACSL1, ACSL3, and ACSL4. ACSL1 expression is decreased in BC [134]. The combination of valproic acid (VPA) and cisplatin modulates lipid metabolism in BC cells by upregulating ACSL1 mRNA expression [135]. In contrast, ACSL1 downregulation has been shown to reduce LPS-induced activation of MAPK and NF-κB signaling pathways, thereby mitigating the inflammatory response in BC [105]. This paradox may stem from the dual roles of ACSL1 in ferroptosis and immune regulation, warranting further investigation. Elevated ACSL3 expression initially inhibits tumor proliferation by increasing LD storage, thereby reducing FAO and cellular energy production. Additionally, ACSL3 promotes tumor cell apoptosis by downregulating phosphorylated YES proto-oncogene (pYES1), reducing nuclear localization of YAP, and decreasing B-cell lymphoma-extra large (BCL-xL) expression [48]. These findings suggest ACSL3 as a potential therapeutic target in BC. During BC progression, ACSL4 acts as a tumor-suppressive regulator through its pro-ferroptotic activity.

Low ACSL4 expression correlates with poor prognosis in triple-negative breast cancer (TNBC) [136]. ACSL4 enhances cancer cell sensitivity to ferroptosis by altering cellular lipid composition, thereby promoting the accumulation of lipid peroxides (LPO) [137]. However, when used as a target for adjuvant radiotherapy, ACSL4 exhibits effects contrary to its role in ferroptosis. Inhibition of ACSL4 can overcome radioresistance by suppressing the DNA damage response, thereby promoting apoptosis in cancer cells during radiotherapy [138]. Additionally, ACSL4 upregulates ATP-binding cassette sub-family G (ABCG2) expression, reducing intracellular doxorubicin accumulation and thereby contributing to chemoresistance [139]. Inhibition of ACSL4 has been shown to enhance chemotherapy efficacy in cancer cells [140]. This paradox highlights the need for further exploration in both preclinical and clinical translational studies.

#### Glioblastoma

ACSL1 and ACSL5 have prognostic significance in gliomas, influencing tumor immunity and immune cell migration, thereby providing valuable insights into low-grade glioma (LGG) prognosis and potential therapeutic targets [141]. Glioblastoma (GBM) has been shown to enhance FA metabolism by upregulating ACSL expression, compensating for the loss of Rest, which is required for tumor growth [142]. In this context, the ACSL family appears to act as a negative regulator, although further research is needed to confirm this observation. ACSL3 promotes tumor progression by enhancing cellular energy metabolism [143]. Inhibition of ACSL4 suppresses tumor invasion by mitigating ferroptosis [144]. Conversely, one study suggests that upregulation of ACSL4 exerts an antiproliferative effect in GBM by promoting ferroptosis [145], thereby positioning ACSL4 and ferroptosis as tumor-protective factors. This paradox raises important questions. It is proposed that the role of ACSL4 and ferroptosis in GBM development is not confined to a single risk or protective pathway. Rather, ACSL4 operates through multifaceted and multi-directional regulatory mechanisms. This complexity presents a significant challenge for future clinical translation. Determining how to balance these roles remains a critical issue, requiring further in-depth investigation in future studies.

#### Other cancers

The ACSL family has also been implicated in the progression of various cancers, including clear cell renal cell carcinoma (ccRCC), oral squamous cell carcinoma (OSCC) and hematologic malignancies.

Low ACSL1 expression has been related with adverse tumor histopathology and reduced overall survival in patients with ccRCC, suggesting its potential as a prognostic marker [146, 147]. ACSL3, in contrast, modulates tumor malignancy through the tumor microenvironment by regulating immune cell abundance. It functions as a tumor-protective factor [148]. ACSL4 contributes to ccRCC progression by inducing lipid peroxidation overload, thereby promoting ferroptosis in ccRCC cells [149]. Significant gaps remain in research on the ACSL family in ccRCC, which warrant further investigation.

In OSCC, m6A demethylation of ACSL3 facilitates its degradation, thereby promoting ferroptosis in OSCC cells [150]. Flap endonuclease 1 (FEN1) binds to the ACSL4 promoter region, leading to suppressing ferroptosis in OSCC cells [151]. Research on the ACSL family in OSCC is limited, with the majority of studies focusing on ferroptosis.

The ACSL family also plays a significant role in hematologic malignancies. Study has identified the ACSL family as a novel therapeutic target in multiple myeloma [152]. Knockdown of ACSL4 suppresses multiple myeloma (MM) cell proliferation and decreases FA levels, potentially through the regulation of lipid metabolism-related genes, including c-Myc and sterol regulatory element-binding proteins (SREBPs) [153]. Knockdown of ACSL4 decreases dihomo-γ-linolenic acid (DGLA)-induced ferroptosis sensitivity in acute myeloid leukemia (AML) cells. ACSL4 reprograms DGLA-associated lipids to induce ferroptosis in AML cells [154, 155]. ACSL1 promotes imatinib-induced senescence and tumor growth in K562 cells via regulation of the SIRT1/p53/p21 pathway. The ACSL1/sirtuin 1 (SIRT1)/p53 signaling axis represents a novel mechanism underlying senescence in chronic myeloid leukemia (CML) cells [156].

ACSL1 promotes endometrial cancer metastasis [157] and drives prostate cancer progression by enhancing FA β-oxidation [158]. At the same time, its expression inhibits ferroptosis, thereby enhancing platinum resistance in ovarian cancer [159]. ACSL4 can attenuate the progression of prostate [160] and ovarian cancers by promoting ferroptosis in cancer cells. In ovarian cancer, ACSL4 also facilitates M1 macrophage polarization, leading to inhibiting tumor progression [161]. Conversely, downregulation of ACSL4 reduces ferroptosis, a process that facilitates the progression of cervical cancer [162]. In addition to the aforementioned pathways involving lipid peroxidation, immunity, and ferroptosis, study has shown that ACSL3 and ACSL4 exert opposing regulatory effects on the androgen receptor (AR), consistent with their contrasting roles in ferroptosis. However, whether this regulatory mechanism is directly related to ferroptosis remains unclear [163].

Notably, deletion of ACSL1 has been observed in cachexia, a phenomenon that has not been previously reported. ACSL1 expression decreases as cachexia progresses [164]. This raises the intriguing question of whether ACSL1 could serve as a potential therapeutic target in cachexia. In addition, it is undeniable that current study reveals a certain degree of contradiction regarding the role of ACSL4, ferroptosis, and cancer, as ACSL4-mediated ferroptosis appears to exert a bidirectional effect in tumor progression [163]. The underlying causes and molecular mechanisms of this bidirectional role require further investigation.

**Fig. 4:**
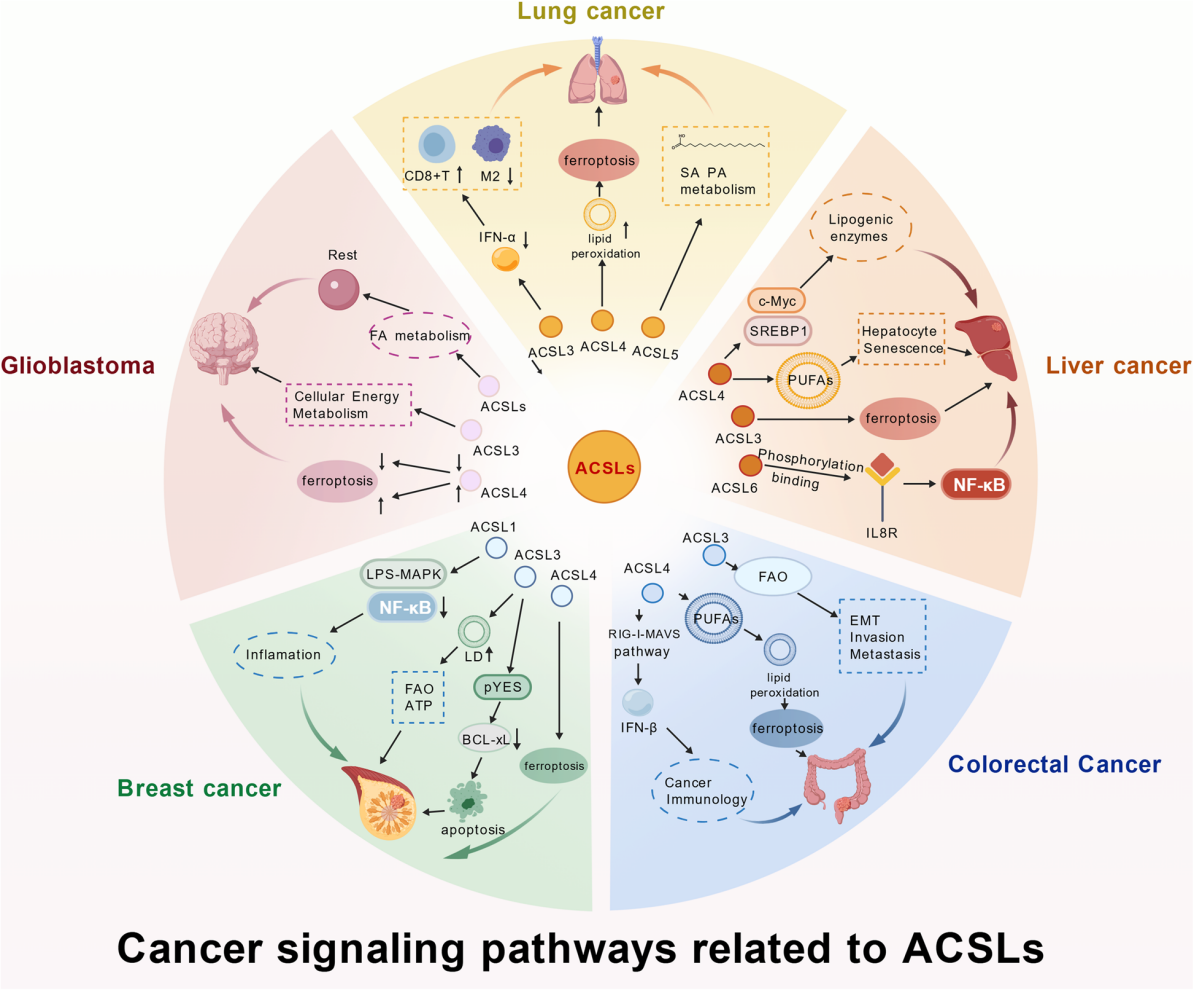
The mechanisms of the ACSL family in cancers. Mechanistic pathways of ACSL involvement in cancers, including lung cancer, liver cancer, colorectal cancer, breast cancer and glioblastoma. ACSL (long-chain acyl-CoA synthetases), M2 (macrophage M2 phenotype), SA (sialic acid), PA (palmitic acid), c-Myc (cellular myelocytomatosis), SREBP1 (sterol regulatory element-binding protein 1), PUFAs (polyunsaturated fatty acids), IL8R (interleukin-8 receptor), NF-κB (nuclear factor kappa-light-chain-enhancer of activated B cells), FAO (fatty acid oxidation), EMT (epithelial-mesenchymal transition), RIG-I (retinoic acid-inducible gene I), MAVS (mitochondrial antiviral-signaling protein), IFN-β (interferon-beta), pYES (phosphorylate YES proto-oncogene), BCL-XL (B-cell lymphoma-extra large), LD (lipid droplet), LPS (lipopolysaccharide), MAPK (mitogen-activated protein kinase)

Based on the discussion in this section, the key pathways through which ACSLs are involved in cancer are summarized in Fig. 4. As illustrated in the figure, ACSLs primarily influence cancer initiation and progression through FA oxidation, ferroptosis, and immune-associated pathways. In addition, Table 2 summarizes the model systems and upstream or downstream targets employed in the aforementioned disease studies.

**Table 2:** ACSLs and diseases, including related signaling ways

Diseases	ACSLs	Upstream	Downstream	Methods	Reference
MAFLD	ACSL1	LCFAs	FAO	in vitro and in vivo experiment	[70]
		-	BA	in vitro and in vivo experiment	[71]
		-	FA consumption	in vitro experiment	[79]
		-	TBK1	in vitro and in vivo experiment	[80]
	ACSL4	-	Mitochondrial respiration, Fatty acid beta oxidation PPARs	in vivo experiment	[74]
	ACSL5	SIRT6	FAO	review	[81]
Diabetes	ACSL1	Sortilin	-	in vitro and in vivo experiment	[83]
	ACSL4	-	ferroptosis	review	[84]
Diabetic nephropathy	ACSL1	-	PPARα PPARγ	in vitro and in vivo experiment	[42]
	ACSL4	-	ferroptosis	in vitro and in vivo experiment	[88]
	ACSL5	-	FATP2	in vitro and in vivo experiment	[89]
Diabetic retinopathy	ACSL4	glucose	-	in vitro and in vivo experiment	[90]
	-	NKAα1	ferroptosis	in vitro and in vivo experiment	[92]
Obesity	ACSL1	-	LCA-CoA Cer DAG	in vivo experiment	[93]
	ACSL4	-	p53 activation	in vitro and in vivo experiment	[94]
	ACSL5	-	Fatty acid β-oxidation	in vitro experiment	[99]
		-	GLP, PYY	in vivo experiment	[96]
	ACSL6	-	Glucose and fatty acid metabolism	in vitro experiment	[100]
Lung cancer	ACSL3	-	ferroptosis	in vitro experiment	[108]
		-	IFN-α	in vitro and in vivo experiment	[109]
	ACSL4	-	ferroptosis	in vitro and in vivo experiment	[110, 111]
	ACSL5	-	transcriptional regulator	in vitro experiment	[114]
		-	Metabolism of palmitic and stearic acids	Clinical Trans-omics	[115]
Liver cancer	ACSL4	-	c-Myc/SREBP1	in vitro and in vivo experiment	[117]
		-	ferroptosis	in vitro experiment	[118]
		-	Fatty acid biosynthesis, lipid peroxidation, TME	in vitro and in vivo experiment	[119]
		-	Liver cell senescence	in vitro and in vivo experiment	[120]
		-	FXR/BA/M2 polarize	in vitro and in vivo experiment	[121]
	ACSL3	-	PPARα	in vitro and in vivo experiment	[124]
	ACSL6	-	NF-κB	in vitro and in vivo experiment	[127]
Colorectal cancer	ACSL3	-	FAO	in vitro and in vivo experiment	[128]
		-	ferroptosis	in vitro and in vivo experiment	[129]
		-	IFN-β	in vitro and in vivo experiment	[131]
Breast cancer	ACSL1	-	lipid metabolism	in vitro experiment	[135]
		-	NF-κB	in vitro experiment	[105]
	ACSL3	-	pYES1	in vitro and in vivo experiment	[48]
	ACSL4	-	ferroptosis	in vitro and in vivo experiment	[137]
		-	ABCG2	in vitro and in vivo experiment	[139]
Glioblastoma	ACSL3	-	cellular energy metabolism	in vitro and in vivo experiment	[143]
	ACSL4	-	ferroptosis	in vitro and in vivo experiment	[144]

*MAFLD *Metabolic dysfunction-associated steatotic liver disease, *ACSL *long-chain acyl-CoA synthetase, *LCFA* long-chain fatty acid, *FAO* fatty acid oxidation, *BA* bile acid, *FA* fatty acid, *TBK1* TANK-binding kinase 1, *SIRT* sirtuin, *PPAR* peroxisome proliferator-activated receptor, *FATP* fatty acid transport protein, *NKAα1* Na^+^/K^+^ ATPase α1 subunit, *LCA-CoA* long-chain acyl-coenzyme A, *Cer* ceramide, *DAG* diacylglycerol, *GLP-1* glucagon-like peptide-1, *PYY* peptide YY, *IFN* interferon, *c-Myc* cellular myelocytomatosis, *SREBP1* sterol regulatory element-binding protein 1, *TME* tumor microenvironment, *FXR* farnesoid X receptor, *NF-κB* nuclear factor kappa-light-chain-enhancer of activated B cells, *pYES* phosphorylate YES proto-oncogene, *ABCG* ATP-binding cassette sub-family G

### Cardiovascular and cerebrovascular diseases

#### Cardiovascular diseases

The role of ACSL1 in cardiovascular disease remains somewhat ambiguous. Nevertheless, ACSL1 has been shown to preserve mitochondrial energy by increasing the availability of acyl-CoA, thus preventing the production of lipotoxic ceramides and mitigating cardiac dysfunction during chronic pressure overload. Overexpression of ACSL1 reduces cardiac hypertrophy and functional decline [165]. In contrast, one study reports that knockdown of ACSL1 effectively restores cardiac function and promotes myocardial regeneration following myocardial infarction [166]. Downregulation of ACSL1 mitigates the pro-inflammatory effects of palmitic acid (PA), thereby alleviating inflammation in cardiovascular disease [167]. ACSL3 is proposed to play a protective role in atherosclerosis, potentially through the regulation of ferroptosis [168]. In contrast, ACSL4, which exerts effects opposite to ACSL3 in ferroptosis, is considered a risk factor in cardiovascular disease. Inhibition of ACSL4 has been shown to protect against cardiac ischemia/reperfusion (I/R) injury by suppressing ferroptosis [169]. Beyond ferroptosis, ACSL4 acts to exacerbate myocardial injury, promoting lipopolysaccharide (LPS)-induced lipid peroxidation [170]. In conclusion, the role of ACSL1 in cardiovascular disease remains to be fully elucidated, including whether it functions as a protective factor, a risk factor, or a bidirectional regulator. In addition to the roles of ACSL3 and ACSL4 in cardiovascular disease via the ferroptosis pathway, further investigation of alternative pathways may provide additional insights into the contributions of these targets to cardiovascular pathology.

#### Cerebrovascular diseases

Among cerebrovascular diseases, the ACSL family—particularly ACSL4—has been extensively studied. ACSL4 expression is markedly upregulated following cerebral I/R injury [171]. Similar upregulation of ACSL4 has been observed in brain injury following subarachnoid hemorrhage (SAH) [172]. Inhibition of ACSL4-mediated ferroptosis alleviates neuroinflammation following SAH [173]. ACSL4 promotes lipid peroxidation by facilitating the conversion of PUFAs to fatty acyl-CoA, thereby inducing neuronal ferroptosis and exacerbating brain injury. ACSL4 thus represents a risk factor in cerebrovascular disease [174]. Inhibition of ACSL4 markedly reduces brain edema [175]. Several studies have investigated ACSL4 as a therapeutic target for ferroptosis, aiming to mitigate neuronal injury by modulating the ACSL4-ferroptosis pathway [176, 177]. While numerous studies have investigated targeting ACSL4 to mitigate brain injury in cerebrovascular disease, the roles of ACSL1 and ACSL3 as key regulators of ferroptosis remain underexplored. Additionally, it remains to be determined whether ACSL4, as a central enzyme in fatty acid metabolism, influences glycolipid metabolism in patients with cerebrovascular disease.

ACSLs play a pivotal role in the development and progression of cardiovascular and cerebrovascular diseases through their regulatory functions in fatty acid metabolism, inflammatory responses, and ferroptosis.

### Other diseases

In addition to the aforementioned disease types, ACSLs also play important roles in several other pathological conditions. This subsection provides an overview of these additional disease contexts.

#### Neurodegenerative disease

ACSL4 contributes to the pathogenesis of Alzheimer’s disease through the promotion of ferroptosis [178]. Inhibition of ACSL4 promotes the differentiation of neural stem cells into neurons, suggesting its potential as a therapeutic target for neurodegenerative diseases [179]. ACSL3 mitigates Alzheimer’ s disease pathology by activating the brain-derived neurotrophic factor (BDNF) and vascular endothelial growth factor C (VEGF-C) signaling pathways, with its underlying mechanism potentially linked to the synthesis of ω−3 and ω−6 FAs [180]. ACSL1 aggravates neuroinflammation in Parkinson’s disease by facilitating lipid droplet biogenesis [181]. Additionally, ACSL4 is upregulated in the substantia nigra of patients with Parkinson’s disease, and its inhibition ameliorates Parkinsonian phenotypes [182]. The zinc finger protein (ZFP36) directly binds to ACSL4 mRNA, suppressing its expression and thus negatively regulating ACSL4-mediated oxidative stress and ferroptosis. This mechanism impacts dopaminergic neurons in Parkinson’s disease, offering potential strategies for its prevention and treatment [183]. ACSL6-mediated DHA metabolism contributes to the maintenance and function of dopaminergic neurons [184]. Given the critical role of the ACSL family in polyunsaturated fatty acid metabolism, it likely plays a significant role in neurological disorders, particularly ACSL6. However, substantial gaps remain in this area, making it a promising avenue for future research.

#### Fibrosis

ACSL4 exacerbates fibrosis in systemic sclerosis, a process driven by the promotion of ferroptosis in inflammatory macrophages [185]. Knockdown of ACSL4 decreases PUFA-containing membrane phospholipids, thereby mitigating lipid peroxidation and protecting against toxin-induced pulmonary fibrosis [186]. ACSL1 expression is upregulated in activated hepatic stellate cells (HSCs) [187]. ACSL4 has been shown to promote the activation of HSCs, thereby contributing to liver fibrosis [188]. Downregulation of ACSL4 prevents renal fibrosis by reducing ferroptosis in renal tubular cells [189]. In contrast, upregulation of ACSL1 mitigates renal tubulointerstitial fibrosis by reducing lipid accumulation [190]. Recent studies on the role of the ACSL family in fibrotic diseases indicate that these enzymes predominantly act through lipid metabolism or ferroptosis. However, whether interactions exist between these two pathways remains largely unexplored. Owing to the close relationship between ferroptosis and lipid metabolism, investigating their interplay represents a promising direction for future research.

#### Organ ischemia–reperfusion injury diseases

ACSL4 is upregulated in the injured kidney [191]. Knockdown of ACSL4 suppresses cellular ferroptosis and inflammatory responses, thereby mitigating kidney injury. These findings suggest that targeting ACSL4 or its regulatory molecules may represent a potential therapeutic approach for acute kidney injury (AKI) [59]. Zhao, Li, et al. demonstrated that the interaction between cytoplasmic high mobility group box 1 (HMGB1) and ACSL4 exacerbates renal injury during AKI [192]. In 2022, Dex et al. demonstrated that inhibition of ACSL4 via the α2-adrenergic receptor (α2-AR) attenuates ferroptosis-mediated renal I/R injury and inflammation [193]. Beyond molecular studies, genetic research has demonstrated that miR-20a-5p may serve as a potential therapeutic agent by inhibiting ACSL4-dependent ferroptosis in renal transplantation [194]. Pulmonary I/R injury is regulated by ACSL4 through modulation of ferroptosis. Therefore, ACSL4 represents a potential therapeutic target for the treatment and prevention of pulmonary I/R injury [195]. Research indicates that ACSL4 plays a significant role in intestinal I/R injury by mediating ferroptosis [196]. Moreover, elevated ACSL4 expression exacerbates hepatic I/R injury. ACSL4 may serve as a potential therapeutic target for hepatic I/R injury [197].

## ACSLs as Therapeutic Targets

Building upon the in-depth elucidation of the pivotal roles of ACSLs in multiple disease pathways, the development of drugs targeting this enzyme family has become a crucial step in advancing related therapies toward clinical translation. Current development strategies have expanded beyond conventional synthetic small-molecule inhibitors to encompass natural product screening, reflecting an increasing trend toward diversification. Regarding synthetic compounds, research has advanced into the mechanisms of classic non-specific inhibitors (e.g., Triacsin C [198]), while highly potent inhibitors with subtype selectivity (e.g., the benzimidazole derivative compound 13 [199]) have emerged, exhibiting favorable pharmacokinetic profiles. Concurrently, numerous natural products (e.g., ergosterol [200], astaxanthin [201], and EGCG [169]) have been shown to exert therapeutic effects in metabolic diseases, cancers, and organ protection by selectively modulating the activity of specific ACSL isoforms. These findings not only provide precise chemical probes for functional studies of the ACSL family but also establish a solid foundation for the development of novel targeted therapeutics. This section systematically reviews recent advances in compound-based research targeting ACSLs, with a focus on their mechanisms of action, subtype specificity, and therapeutic potential.

### Synthetic agents​

#### Triacsin C

Triacsin C is a well-established inhibitor of the ACSL family, exerting a non-specific inhibitory effect on ACSL isoforms and modulating cellular lipid metabolism [202]. By inhibiting ACSL activity, triacsin C can impact neutrophil function, thereby influencing neutrophil responses [198]. However, whether this translates into a role in immunomodulation remains unclear, as no subsequent studies have explored this aspect in depth. More recent research suggests that Triacsin C, at sub-half-maximal inhibitory concentration (IC_50_) (4.1 μM) concentrations, can shift hepatocytes toward a more oxidized state, enhancing FA utilization, accelerating lipid oxidation, and ameliorating MAFLD during long-term treatment [203]. Combined treatment with DHA and Triacsin C significantly induces apoptosis in RL95-2 endometrial cancer cells [204]. Additionally, triacsin C when combined with ABT-199 (a B-cell lymphoma 2 [BCL-2] inhibitor approved by the U.S. Food and Drug Administration [FDA] for treating AML), strongly suppresses cell proliferation and induces apoptosis [205]. These findings highlight triacsin C's potential as a therapeutic agent for both cancer and metabolic disorders, suggesting that expanding its therapeutic indications could be a promising avenue for future research.

#### Benzimidazole series

A recent study identified a series of benzimidazoles as the first highly potent and selective ACSL1 inhibitors through structure–activity relationship (SAR) studies following high-throughput screening of hit compound 1. Among these, the representative analogue 13 exhibited exceptional biological activity. Compound 13 demonstrated IC_50_ values of 0.042 µM and 0.030 µM against human and mouse ACSL1, respectively, while maintaining IC_50_ values > 200 µM against other ACSL isoforms (ACSL3, ACSL4, ACSL5, and ACSL6) in vitro enzymatic assays, indicating remarkable selectivity. The enzymatic activity assays were conducted with recombinant ACSL enzymes in a buffer system containing ATP, coenzyme A, and the corresponding FA substrate, with acyl-CoA production detected by MALDI-TOF MS. In a 3T3-L1 cell-based assay, compound 13 inhibited TG synthesis with an IC_30_ of 0.039 µM. Furthermore, in vivo experiments in mice confirmed its dose-dependent suppression of multiple long-chain acyl-CoA species. Compound 13 demonstrated favorable microsomal stability (96%, 93%, and 99% remaining in human, rat, and mouse microsomes, respectively), negligible CYP inhibition (IC_50_ > 20 µM for CYP1A2, 2C19, 2C9, 2D6, and 3A4), and desirable pharmacokinetic properties (oral bioavailability F = 119% in rats). The benzimidazole analog 13 thus emerged as a potent tool compound for investigating ACSL1’s physiological functions. The study also provided a synthetic route for compound 13, where the benzimidazole core was constructed through a Mitsunobu reaction, methylation, reduction, and cyclization. These findings lay a significant foundation for further exploration of ACSL1 as a therapeutic target and offer new directions for the treatment of metabolic diseases [199].

### Naturally derived compounds and TCM recipe

#### Ergosterol

Ergosterol (ES) specifically targets ACSL1 as its site of action, activating ACSL1 in a concentration-dependent manner with an EC_50_ of 4 μM. Treatment with ES increased ACSL1 activity by approximately twofold in both cell lysates and isolated mitochondria. ES is primarily localized in mitochondria, where it activates ACSL1 to enhance FAO in hepatocytes, thereby exerting lipid-lowering effects. Molecular docking analyses further revealed that ES functions as a selective allosteric agonist of ACSL1. The study confirmed, using multiple complementary techniques—including activity-based protein profiling, microscale thermophoresis, and surface plasmon resonance—that ES binds directly to the ACSL1 protein, with a dissociation constant (K_d) of approximately 2–6 μM, indicating a strong binding affinity [200]. These findings suggest that ACSL1 serves as a lipid-lowering target for ES.

#### Astaxanthin

Astaxanthin exerts its inhibitory effect on ferroptosis through ACSL4. Molecular docking analysis demonstrated that astaxanthin forms a hydrogen bond with the LEU691 residue of ACSL4 (binding energy: −8.69 kcal/mol) and further stabilizes the complex through hydrophobic interactions, thereby directly suppressing ACSL4 enzymatic activity [201]. While the study clearly showed that astaxanthin mitigates PFOS-induced ferroptosis by targeting ACSL4, it did not provide the IC_50_ from dose-response experiments, such as cell viability assays or lipid peroxidation (MDA) measurements. The 40 μM concentration used in the study produced a significant protective effect, providing a preliminary reference for the effective concentration range. To accurately quantify the efficacy of astaxanthin, future studies should establish concentration gradients and construct dose-response curves to determine IC_50_ values for its action on the ferroptosis pathway. Such data will be critical for developing astaxanthin as a dose-defined pharmacological inhibitor of ferroptosis.

#### Other naturally derived compounds

Numerous natural products have been shown to modulate ACSL family members. Epigallocatechin gallate (EGCG) suppresses ferroptosis via the miR-450b-5p/ACSL4 axis, thereby mitigating myocardial ischemic injury. The results of this study demonstrated that administration of either 10 mg/kg or 20 mg/kg EGCG significantly ameliorated myocardial injury in mice [169]. Capsaicin inhibits ferroptosis by promoting ACSL4 ubiquitination. The study revealed that capsaicin at concentrations ranging from 0.1 to 1 μM promoted cell proliferation, while concentrations between 10 and 100 μM exhibited an inhibitory effect [206]. Vaccarin mitigates renal fibrosis by suppressing ferroptosis through downregulation of ACSL4. The study indicated that administering Vaccarin at doses of 12.5 mg/kg and 25 mg/kg elicited a favorable therapeutic response in the mouse model [207]. Nobiletin may exert therapeutic effects in type 2 diabetes by downregulating ACSL4, with the study confirming that administration of the drug at a dose of 5 mg/kg yielded a beneficial effect in the rat model [208]. Apigenin upregulates ACSL1 expression and enhances FFA β-oxidation, thereby reducing intracellular lipid accumulation. The study confirmed that apigenin exerted no cytotoxic effect on cell viability within the concentration range of 2.5 to 20 μM [209]. Terpenoids have been reported to modulate ferroptosis by inhibiting ACSL4 activity [210]. One study indicated that ACSL4 may serve as a potential target protein for geraniol. Additionally, puerarin at a concentration of 40 μM effectively alleviated inflammation [211]. While numerous natural products target the ACSL family, further exploration is needed to address challenges related to isoform specificity.

The preceding section summarizes recent advances in the development of compounds targeting ACSLs. The relevant information is compiled in Table 3.

**Table 3:** Preclinical studies related to ACSLs

chemical compound	ACSL	Direction of action	Mechanism	Experimental model	disease	References
Triacsin C	ACSLs	inhibition	Fatty acid metabolism	in vivo and in vitro model	MAFLD	[203]
Benzimidazole series	ACSL1	inhibition	-	in vivo model	-	[199]
Ergosterol	ACSL1	activation	Fatty acid oxidation	in vivo and in vitro model	hepatic steatosis	[200]
Epigallocatechin gallate	ACSL4	downregulation	miR-450b-5p/ACSL4 axis	in vivo and in vitro model	myocardial ischemic injury	[169]
Capsaicin	ACSL4	downregulation	ferroptosis	in vitro model	CRC	[206]
Nobiletin	ACSL4	downregulation	ferroptosis	in vivo and in vitro model	diabetes	[208]
Apigenin	ACSL1	upregulation	Fatty acid β oxidation	in vitro model	MAFLD	[209]
Astaxanthin	ACSL4	inhibition	ferroptosis,lipid accumulation,cell injury	in vivo and in vitro model	liver lipid metabolism disorders	[201]

*ACSL* long-chain acyl-CoA synthetase, *MAFLD* metabolic dysfunction -associated steatotic liver disease, *CRC* colorectal cancer

#### TCM recipe

In addition to isolated natural products, traditional Chinese medicine (TCM) formulas play an important role in traditional therapeutics. These formulas are characterized by multi-target and multi-pathway mechanisms, and elucidating their primary modes of action remains a key challenge. Existing studies suggest that TCM formulas can exert effects through the ACSL family and associated signaling pathways. For example, HJ11 decoction has been shown to attenuate myocardial I/R injury by inhibiting ACSL4-mediated ferritin deposition [212]. Gancao Xiexin decoction attenuates ferroptosis in ulcerative colitis (UC) via the TEA domain transcription factor 4 (TEAD4)/ACSL4 signaling pathway [213].

## Summary and future perspectives

ACSL enzymes are central to lipid metabolism and FAO, playing key roles in various physiological and pathological processes, including cellular metabolism, ER stress, ferroptosis, and tissue inflammatory responses. Ferroptosis, in particular, has become a major area of recent research. Among the ACSL family, ACSL1, ACSL3, and ACSL4 are especially critical to ferroptosis. These enzymes influence systemic glycolipid metabolism and local FFA accumulation through FAO, lipid metabolism, inflammatory responses, immune signaling pathways, and ferroptosis. As such, ACSL enzymes are implicated in cancer, metabolic disorders, cardiovascular and cerebrovascular diseases, and neurodegenerative pathologies, positioning them as potential biomarkers and therapeutic targets. However, their therapeutic applications, drug development, and clinical translation warrant further investigation.

Mechanistic studies of metabolic diseases have primarily focused on how ACSL enzymes regulate lipid metabolic flux and biosynthetic pathways, affecting systemic energy homeostasis and metabolic balance. In cancer research, attention has been directed to their roles in lipid peroxidation, oxidative stress, and ferroptosis—factors that are critical for tumor progression and therapeutic resistance. Similarly, studies on cardiovascular diseases have examined how ACSL family members regulate lipid metabolism, inflammatory signaling, and vascular pathophysiology.

These studies indicate a shift from characterizing the structural and functional diversity of ACSL isoforms to understanding the molecular signaling pathways through which they contribute to disease initiation and progression. As research progresses, mechanistic insights have become more refined. Nevertheless, significant knowledge gaps remain, emphasizing the need for systematic, interdisciplinary investigations to fully elucidate the biological and clinical significance of the ACSL family.

Looking forward, several key research directions in the study of the ACSL family warrant attention. First, while substantial progress has been made in understanding the role of ACSL4 in ferroptosis, the functions of other isoforms such as ACSL1 and ACSL3, which are also important metabolic regulators, have been less explored. Their potential involvement in ferroptosis-related diseases could be clarified through integrative analyses of clinical samples and multi-omics approaches, including transcriptomics, proteomics, and metabolomics. These studies would help define the specific regulatory networks in which these isoforms participate. Additionally, it remains unclear whether certain ACSL isoforms may exert antagonistic or compensatory effects within ferroptosis—an area that warrants detailed mechanistic and functional validation.

Second, the role of ACSL isoforms in tumorigenesis and cancer progression remains insufficiently characterized. While ACSL4 has been extensively studied in relation to lipid peroxidation and ferroptotic sensitivity in tumors, other family members have not been comprehensively evaluated. For example, ACSL6, a brain-enriched isoform with critical roles in neuronal lipid metabolism and synaptic function, was not included in our current study but may hold significant potential as a therapeutic target in GBM and other neurological malignancies. Future studies focusing on the expression patterns, regulatory mechanisms, and downstream effects of these isoforms in various tumor types could provide valuable insights into their clinical relevance.

Thrid, in the context of metabolic diseases, research should extend beyond the canonical functions of ACSLs in FA activation and lipid metabolism to include their roles in inflammatory signaling and immune modulation. As ACSL family members act as regulatory hubs connecting metabolic and inflammatory pathways, they may serve as pivotal mediators that coordinate multiple disease-related processes. Therefore, targeting specific ACSL isoforms could potentially produce dual therapeutic effects, simultaneously modulating metabolic balance and inflammatory responses.

Finally, although numerous inhibitors targeting the ACSL family have been identified, they remain at the preclinical stage. The translation of these therapeutically promising compounds into clinical trials constitutes an important direction for future research.

Advancing the understanding of these unexplored aspects of the ACSL family will deepen our insights into their multifaceted biological functions and lay the foundation for precision therapeutic strategies that integrate both metabolic and immunological regulation.

## Supplementary Information

Supplementary Material 1.

## Data Availability

Not applicable.

## Abbreviations

FFA: Free fatty acid
SCFA: Short-chain fatty acid
MCFA: Medium-chain fatty acid
LCFA: Long-chain fatty acid
ACS: Acyl-CoA synthetase
FA: Fatty acid
ACSL: Acyl-CoA synthetase long-chain
ER: Endoplasmic reticulum
AMP: Adenosine monophosphate
PPAR: Peroxisome proliferator-activated receptor
CDX2: Caudal type homeobox 2
HUFA: Highly unsaturated fatty acids
DHA: Docosahexaenoic acid
EPA: Eicosapentaenoic acid
AA: Arachidonic acid
LD: Lipid droplet
TG: Triglyceride
PL: Phospholipid
ACSL4KO: Acyl-CoA synthetase long-chain family member 4 knockout
CPT1b: Carnitine palmitoyltransferase 1b
PUFA: Polyunsaturated fatty acid
LPS: Lipopolysaccharide
GM-CSF: Granulocyte-macrophage colony-stimulating factor
MAPK: Mitogen-activated protein kinase
NF-κB: Nuclear factor kappa-light-chain-enhancer of activated B cells
αESA: α-Eleostearic acid
FSP1: Ferroptosis suppressor protein 1
CoQ10: Coenzyme Q10
MUFA: Monounsaturated fatty acid
LD: Lipid droplet
BC: Breast cancer
PUFA-PLs: Polyunsaturated fatty acid-containing phospholipids
ROS: Reactive oxygen species
rPMACs: Resident peritoneal macrophages
DC: Dendritic cell
MitoSOX: Mitochondrial superoxide indicator
MHC-I: Major histocompatibility complex class I
PD-1: Programmed cell death protein 1
ULC-PUFA: Ultra-long-chain polyunsaturated fatty acid
MAFLD: Metabolic dysfunction-associated fatty liver disease
FAO: Fatty acid oxidation
MASH: Metabolic dysfunction-associated steatohepatitis
HFD: High-fat diet
5-HETE: 5-Hydroxyeicosatetraenoic acid
TANK: TRAF family member-associated NF-κB activator
TBK1: TANK-binding kinase 1
T2DM: Type 2 diabetes mellitus
PRMT: Protein arginine methyltransferase
FATP2: Fatty acid transport protein 2
ALKBH5: AlkB homolog 5
NKAα1: Na^+^/K^+^ ATPase α1 subunit
LCA-CoA: Long-chain acyl-coenzyme A
Cer: Ceramide
DAG: Diacylglycerol
TA: Traumatic acid
GLP-1: Glucagon-like peptide-1
PYY: Peptide YY
STAT3: Signal transducer and activator of transcription 3
NLRCS: NOD-like receptor family CARD domain containing 5
IFN: Interferon
LPCAT3: Lysophosphatidylcholine acyltransferase 3
YAP: Yes-associated protein
NSCLC: Non-small cell lung cancer
HCC: Hepatocellular carcinoma
c-Myc: Cellular myelocytomatosis
SREBP1: Sterol regulatory element-binding protein 1
FXR: Farnesoid X receptor
M2: Macrophage M2 phenotype
PORS: Progesterone receptor
ERK2: Extracellular signal-regulated kinase 2
IL18R: Interleukin-18 receptor
EMT: Epithelial-mesenchymal transition
CRC: Colorectal cancer
RIG-I: Retinoic acid-inducible gene I
MAVS: Mitochondrial antiviral-signaling protein
IFN-β: Interferon-beta
pYES1: Phosphorylate YES proto-oncogene
BCL-xL: B-cell lymphoma-extra large
TNBC: Triple-negative breast cancer
ABCG: ATP-binding cassette sub-family G
GBM: Glioblastoma
LGG: Lower-grade glioma
CEROC: Ceramide oxidase
OSCC: Oral squamous cell carcinoma
FEN1: Flap endonuclease 1
MM: Multiple myeloma
DGLA: Dihomo-γ-linolenic acid
AML: Acute myeloid leukemia
SIRT1: Sirtuin 1
CML: Chronic myeloid leukemia
AR: Androgen receptor
PA: Palmitic acid
I/R: Ischemia-reperfusion
SAH: Subarachnoid hemorrhage
BDNF: Brain-derived neurotrophic factor
VEGF-C: Vascular endothelial growth factor C
ZFP: Zinc finger protein
HSC: Hepatic stellate cell
AKI: Acute kidney injury
HMGB1: High mobility group box 1
α2-AR: Alpha-2 adrenergic receptor
BCL-2: B-cell lymphoma 2
FDA: U.S. Food and Drug Administration
TCM: Traditional Chinese medicine
UC: Ulcerative colitis
TEAD4: TEA domain transcription factor 4
