# Supplementary material for: Long-chain acyl-CoA synthetases: biological functions, diseases and therapeutic targets
Source: Mol Biomed. 2025 Nov 25;6:117. doi: 10.1186/s43556-025-00366-4 (PMC12647431; doi:10.1186/s43556-025-00366-4)
Supplement: Supplementary file 1 — Supplementary Material 1. [file 43556_2025_366_MOESM1_ESM.docx]

**Supplementary Materials**

The protein sequences of human ACSL1, ACSL3, ACSL4, ACSL5, ACSL6 from the Unified Protein Data Bank are described as follows.

1. **Long-chain-fatty-acid--CoA ligase 1·Homo sapiens (Human)**
2. **Long-chain-fatty-acid--CoA ligase 3·[Homo sapiens (Human)](https://www.uniprot.org/taxonomy/9606" \o "Homo sapiens (Human), taxon ID 9606)**
3. **Long-chain-fatty-acid--CoA ligase 4·Homo sapiens (Human)**
4. **Long-chain-fatty-acid--CoA ligase 5·Homo sapiens (Human)**
5. **Long-chain-fatty-acid--CoA ligase 6·Homo sapiens (Human)**
6. **Long-chain-fatty-acid--CoA ligase 1·Homo sapiens (Human)**

**amino acid sequence:**

>sp|P33121|ACSL1_HUMAN Long-chain-fatty-acid--CoA ligase 1 OS=Homo sapiens OX=9606 GN=ACSL1 PE=1 SV=1

MQAHELFRYFRMPELVDFRQYVRTLPTNTLMGFGAFAALTTFWYATRPKPLKPPCDLSMQ

SVEVAGSGGARRSALLDSDEPLVYFYDDVTTLYEGFQRGIQVSNNGPCLGSRKPDQPYEW

LSYKQVAELSECIGSALIQKGFKTAPDQFIGIFAQNRPEWVIIEQGCFAYSMVIVPLYDT

LGNEAITYIVNKAELSLVFVDKPEKAKLLLEGVENKLIPGLKIIVVMDAYGSELVERGQR

CGVEVTSMKAMEDLGRANRRKPKPPAPEDLAVICFTSGTTGNPKGAMVTHRNIVSDCSAF

VKATENTVNPCPDDTLISFLPLAHMFERVVECVMLCHGAKIGFFQGDIRLLMDDLKVLQP

TVFPVVPRLLNRMFDRIFGQANTTLKRWLLDFASKRKEAELRSGIIRNNSLWDRLIFHKV

QSSLGGRVRLMVTGAAPVSATVLTFLRAALGCQFYEGYGQTECTAGCCLTMPGDWTAGHV

GAPMPCNLIKLVDVEEMNYMAAEGEGEVCVKGPNVFQGYLKDPAKTAEALDKDGWLHTGD

IGKWLPNGTLKIIDRKKHIFKLAQGEYIAPEKIENIYMRSEPVAQVFVHGESLQAFLIAI

VVPDVETLCSWAQKRGFEGSFEELCRNKDVKKAILEDMVRLGKDSGLKPFEQVKGITLHP

ELFSIDNGLLTPTMKAKRPELRNYFRSQIDDLYSTIKV

**Human ACSL1:**

**Seq Identity**

100.00%

Template

[P33121.1.A](https://swissmodel.expasy.org/repository/uniprot/P33121?model=AF-P33121-F1-model-v4) Long-chain-fatty-acid--CoA ligase 1
AlphaFold DB model of ACSL1_HUMAN (gene: ACSL1, organism: Homo sapiens (Human))

1. **Long-chain-fatty-acid--CoA ligase 3·[Homo sapiens (Human)](https://www.uniprot.org/taxonomy/9606" \o "Homo sapiens (Human), taxon ID 9606)**

**amino acid sequence:**

>sp|O95573|ACSL3_HUMAN Fatty acid CoA ligase Acsl3 OS=Homo sapiens OX=9606 GN=ACSL3 PE=1 SV=3

MNNHVSSKPSTMKLKHTINPILLYFIHFLISLYTILTYIPFYFFSESRQEKSNRIKAKPV

NSKPDSAYRSVNSLDGLASVLYPGCDTLDKVFTYAKNKFKNKRLLGTREVLNEEDEVQPN

GKIFKKVILGQYNWLSYEDVFVRAFNFGNGLQMLGQKPKTNIAIFCETRAEWMIAAQACF

MYNFQLVTLYATLGGPAIVHALNETEVTNIITSKELLQTKLKDIVSLVPRLRHIITVDGK

PPTWSEFPKGIIVHTMAAVEALGAKASMENQPHSKPLPSDIAVIMYTSGSTGLPKGVMIS

HSNIIAGITGMAERIPELGEEDVYIGYLPLAHVLELSAELVCLSHGCRIGYSSPQTLADQ

SSKIKKGSKGDTSMLKPTLMAAVPEIMDRIYKNVMNKVSEMSSFQRNLFILAYNYKMEQI

SKGRNTPLCDSFVFRKVRSLLGGNIRLLLCGGAPLSATTQRFMNICFCCPVGQGYGLTES

AGAGTISEVWDYNTGRVGAPLVCCEIKLKNWEEGGYFNTDKPHPRGEILIGGQSVTMGYY

KNEAKTKADFFEDENGQRWLCTGDIGEFEPDGCLKIIDRKKDLVKLQAGEYVSLGKVEAA

LKNLPLVDNICAYANSYHSYVIGFVVPNQKELTELARKKGLKGTWEELCNSCEMENEVLK

VLSEAAISASLEKFEIPVKIRLSPEPWTPETGLVTDAFKLKRKELKTHYQADIERMYGRK

**Human ACSL3:**

**Seq Identity**

100.00%

Template

O95573.1.A Fatty acid CoA ligase Acsl3

AlphaFold DB model of ACSL3_HUMAN (gene: ACSL3, organism: Homo sapiens (Human))

1. **Long-chain-fatty-acid--CoA ligase 4·Homo sapiens (Human)**

**amino acid sequence:**

>sp|O60488|ACSL4_HUMAN Long-chain-fatty-acid--CoA ligase 4 OS=Homo sapiens OX=9606 GN=ACSL4 PE=1 SV=2

MKLKLNVLTIILLPVHLLITIYSALIFIPWYFLTNAKKKNAMAKRIKAKPTSDKPGSPYR

SVTHFDSLAVIDIPGADTLDKLFDHAVSKFGKKDSLGTREILSEENEMQPNGKVFKKLIL

GNYKWMNYLEVNRRVNNFGSGLTALGLKPKNTIAIFCETRAEWMIAAQTCFKYNFPLVTL

YATLGKEAVVHGLNESEASYLITSVELLESKLKTALLDISCVKHIIYVDNKAINKAEYPE

GFEIHSMQSVEELGSNPENLGIPPSRPTPSDMAIVMYTSGSTGRPKGVMMHHSNLIAGMT

GQCERIPGLGPKDTYIGYLPLAHVLELTAEISCFTYGCRIGYSSPLTLSDQSSKIKKGSK

GDCTVLKPTLMAAVPEIMDRIYKNVMSKVQEMNYIQKTLFKIGYDYKLEQIKKGYDAPLC

NLLLFKKVKALLGGNVRMMLSGGAPLSPQTHRFMNVCFCCPIGQGYGLTESCGAGTVTEV

TDYTTGRVGAPLICCEIKLKDWQEGGYTINDKPNPRGEIVIGGQNISMGYFKNEEKTAED

YSVDENGQRWFCTGDIGEFHPDGCLQIIDRKKDLVKLQAGEYVSLGKVEAALKNCPLIDN

ICAFAKSDQSYVISFVVPNQKRLTLLAQQKGVEGTWVDICNNPAMEAEILKEIREAANAM

KLERFEIPIKVRLSPEPWTPETGLVTDAFKLKRKELRNHYLKDIERMYGGK

**Human ACSL4:**

**Seq Identity**

100.00%

Template

O60488.1.A Long-chain-fatty-acid--CoA ligase 4

AlphaFold DB model of ACSL4_HUMAN (gene: ACSL4, organism: Homo sapiens (Human))

1. **Long-chain-fatty-acid--CoA ligase 5·Homo sapiens (Human)**

**amino acid sequence:**

>sp|Q9ULC5|ACSL5_HUMAN Long-chain-fatty-acid--CoA ligase 5 OS=Homo sapiens OX=9606 GN=ACSL5 PE=1 SV=1

MLFIFNFLFSPLPTPALICILTFGAAIFLWLITRPQPVLPLLDLNNQSVGIEGGARKGVS

QKNNDLTSCCFSDAKTMYEVFQRGLAVSDNGPCLGYRKPNQPYRWLSYKQVSDRAEYLGS

CLLHKGYKSSPDQFVGIFAQNRPEWIISELACYTYSMVAVPLYDTLGPEAIVHIVNKADI

AMVICDTPQKALVLIGNVEKGFTPSLKVIILMDPFDDDLKQRGEKSGIEILSLYDAENLG

KEHFRKPVPPSPEDLSVICFTSGTTGDPKGAMITHQNIVSNAAAFLKCVEHAYEPTPDDV

AISYLPLAHMFERIVQAVVYSCGARVGFFQGDIRLLADDMKTLKPTLFPAVPRLLNRIYD

KVQNEAKTPLKKFLLKLAVSSKFKELQKGIIRHDSFWDKLIFAKIQDSLGGRVRVIVTGA

APMSTSVMTFFRAAMGCQVYEAYGQTECTGGCTFTLPGDWTSGHVGVPLACNYVKLEDVA

DMNYFTVNNEGEVCIKGTNVFKGYLKDPEKTQEALDSDGWLHTGDIGRWLPNGTLKIIDR

KKNIFKLAQGEYIAPEKIENIYNRSQPVLQIFVHGESLRSSLVGVVVPDTDVLPSFAAKL

GVKGSFEELCQNQVVREAILEDLQKIGKESGLKTFEQVKAIFLHPEPFSIENGLLTPTLK

AKRGELSKYFRTQIDSLYEHIQD

**Human ACSL5:**

**Seq Identity**

100.00%

Template

Q9ULC5.1.A Long-chain-fatty-acid--CoA ligase 5

AlphaFold DB model of ACSL5_HUMAN (gene: ACSL5, organism: Homo sapiens (Human))

1. **Long-chain-fatty-acid--CoA ligase 6·Homo sapiens (Human)**

**amino acid sequence:**

>sp|Q9UKU0|ACSL6_HUMAN Long-chain-fatty-acid--CoA ligase 6 OS=Homo sapiens OX=9606 GN=ACSL6 PE=1 SV=4

MQTQEILRILRLPELGDLGQFFRSLSATTLVSMGALAAILAYWFTHRPKALQPPCNLLMQ

SEEVEDSGGARRSVIGSGPQLLTHYYDDARTMYQVFRRGLSISGNGPCLGFRKPKQPYQW

LSYQEVADRAEFLGSGLLQHNCKACTDQFIGVFAQNRPEWIIVELACYTYSMVVVPLYDT

LGPGAIRYIINTADISTVIVDKPQKAVLLLEHVERKETPGLKLIILMDPFEEALKERGQK

CGVVIKSMQAVEDCGQENHQAPVPPQPDDLSIVCFTSGTTGNPKGAMLTHGNVVADFSGF

LKVTEKVIFPRQDDVLISFLPLAHMFERVIQSVVYCHGGRVGFFQGDIRLLSDDMKALCP

TIFPVVPRLLNRMYDKIFSQANTPLKRWLLEFAAKRKQAEVRSGIIRNDSIWDELFFNKI

QASLGGCVRMIVTGAAPASPTVLGFLRAALGCQVYEGYGQTECTAGCTFTTPGDWTSGHV

GAPLPCNHIKLVDVEELNYWACKGEGEICVRGPNVFKGYLKDPDRTKEALDSDGWLHTGD

IGKWLPAGTLKIIDRKKHIFKLAQGEYVAPEKIENIYIRSQPVAQIYVHGDSLKAFLVGI

VVPDPEVMPSWAQKRGIEGTYADLCTNKDLKKAILEDMVRLGKESGLHSFEQVKAIHIHS

DMFSVQNGLLTPTLKAKRPELREYFKKQIEELYSISM

**Human ACSL6:**

**Seq Identity**

100.00%

Template

Q9UKU0.1.A Long-chain-fatty-acid--CoA ligase 6

AlphaFold DB model of ACSL6_HUMAN (gene: ACSL6, organism: Homo sapiens (Human))
